# Supplementary figures and images for: Informed consent rates for neonatal randomized controlled trials in low- and lower middle-income versus high-income countries: A systematic review
Source: PLoS One. 2021 Mar 9;16(3):e0248263. doi: 10.1371/journal.pone.0248263 (PMC7943024; doi:10.1371/journal.pone.0248263)

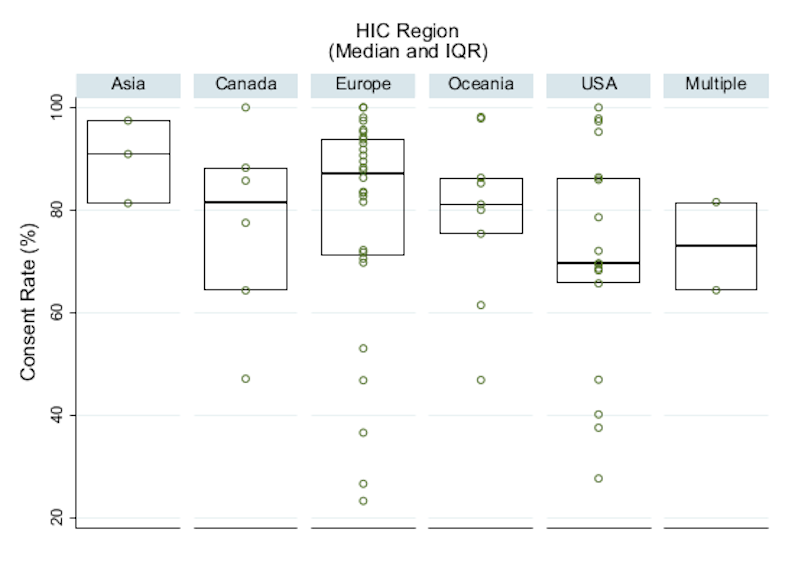

Supplement: S1 Fig — There is no significant difference between median consent rates by region in HIC (p = 0.5087; Kruskal-Wallis test, excludes ‘multiple’). (TIF) [file pone.0248263.s001.tif]
