# Supplementary material for: Informed consent rates for neonatal randomized controlled trials in low- and lower middle-income versus high-income countries: A systematic review
Source: PLoS One. 2021 Mar 9;16(3):e0248263. doi: 10.1371/journal.pone.0248263 (PMC7943024; doi:10.1371/journal.pone.0248263)
Supplement: S1 Table — (DOCX) [file pone.0248263.s002.docx]

**S1 Table. Search strategy for low- and lower middle-income and high-income country articles.**

| **LMIC Search** | | | | |
| --- | --- | --- | --- | --- |
| **Database searched** | **Date searched** | **Search Strategy** | **Filters** | **Results** |
| Pubmed | April 1, 2018 | ((infant, newborn[mesh] OR infant[mesh] OR newborn* OR neonat* OR baby OR babies OR infant*)) AND (“Angola” OR “Armenia” OR “Bangladesh” OR “Bhutan” OR “Bosnia and Herzegovina” OR “Cabo Verde” OR “Cambodia” OR “Cameroon” OR “Congo, Rep.” OR “Cote d’Ivoire” OR “Djibouti” OR “Egypt” OR “El Salvador” OR “Georgia” OR “Ghana” OR “Guatemala” OR “Honduras” OR “India” OR “Indonesia” OR “Jordan” OR “Kenya” OR “Kiribati” OR “Kosovo” OR “Kyrgyz Republic” OR “Lao PDR” OR “Lesotho” OR “Mauritania” OR “Micronesia” OR “Moldova” OR “Mongolia” OR “Morocco” OR “Myanmar” OR “Nicaragua” OR “Nigeria” OR “Pakistan” OR “Papua New Guinea” OR “Philippines” OR “São Tomé and Principe” OR “Solomon Islands” OR “Sri Lanka” OR “Swaziland” OR “Syrian Arab Republic” OR “Tajikistan” OR “Timor-Leste” OR “Tunisia” OR “Ukraine” OR “Uzbekistan” OR “Vanuatu” OR “West Bank and Gaza” OR “Yemen” OR “Afghanistan” OR “Benin” OR “Burkina Faso” OR “Burundi” OR “Central African Republic” OR “Chad” OR “Comoros” OR “Democratic Republic of Congo” OR “Eritrea” OR “Ethiopia” OR “Gambia” OR “Guinea” OR “Guinea-Bissau” OR “Haiti” OR “Democratic People’s Republic of Korea” OR “Liberia” OR “Madagascar” OR “Malawi” OR “Mali” OR “Mozambique” OR “Nepal” OR “Niger” OR “Rwanda” OR “Senegal” OR “Sierra Leone” OR “Somalia” OR “South Sudan” OR “Tanzania” OR “Togo” OR “Uganda” OR “Zimbabwe” OR "low income country" OR "low income countries" OR Deprived Countries[tw] OR Deprived Population[tw] OR Deprived Populations[tw] OR Developing Countries[tw] OR Developing Country[tw] OR Developing Economies[tw] OR Developing Economy[tw] OR Developing Nation[tw] OR Developing Nations[tw] OR Developing Population[tw] OR Developing Populations[tw] OR Developing World[tw] OR LAMI Countries[tw] OR LAMI Country[tw] OR Less Developed Countries[tw] OR Less Developed Country[tw] OR Less Developed Economies [tw] OR Less Developed Nation[tw] OR Less Developed Nations[tw] OR Less Developed World[tw] OR Lesser Developed Countries[tw] OR Lesser Developed Nations[tw] OR LMIC[tw] OR LMICS[tw] OR Low GDP[tw] OR Low GNP[tw] OR Low Gross Domestic[tw] OR Low Gross National[tw] OR Low Income Countries[tw] OR Low Income Country[tw] OR Low Income Economies [tw] OR Low Income Economy[tw] OR Low Income Nations[tw] OR Low Income Population[tw] OR Low Income Populations[tw] OR Lower GDP[tw] OR lower gross domestic[tw] OR Lower Income Countries[tw] OR Lower Income Country[tw] OR Lower Income Nations[tw] OR Lower Income Population[tw] OR Lower Income Populations[tw] OR Middle Income Countries[tw] OR Middle Income Country[tw] OR Middle Income Economies [tw] OR Middle Income Nation[tw] OR Middle Income Nations[tw] OR Middle Income Population[tw] OR Middle Income Populations[tw] OR Poor Countries[tw] OR Poor Country[tw] OR Poor Economies [tw] OR Poor Economy[tw] OR Poor Nation[tw] OR Poor Nations[tw] OR Poor Population[tw] OR Poor Populations[tw] OR poor world[tw] OR Poorer Countries[tw] OR Poorer Economies [tw] OR Poorer Economy[tw] OR Poorer Nations[tw] OR Poorer Population[tw] OR Poorer Populations[tw] OR Third World[tw] OR Transitional Countries[tw] OR Transitional Country[tw] OR Transitional Economies[tw] OR Transitional Economy[tw] OR Under Developed Countries[tw] OR Under Developed Country[tw] OR under developed nations[tw] OR Under Developed World[tw] OR Under Served Population[tw] OR Under Served Populations[tw] OR Underdeveloped Countries[tw] OR Underdeveloped Country[tw] OR underdeveloped economies[tw] OR underdeveloped nations[tw] OR underdeveloped population[tw] OR Underdeveloped World[tw] OR Underserved Countries[tw] OR Underserved Nations[tw] OR Underserved Population[tw] OR Underserved Populations[tw]) AND (randomized controlled trial[pt] OR rct[tiab] OR rcts[tiab] OR "randomized controlled trial" OR "randomized controlled trials" OR "randomized control trial" OR "Randomized control trials") | Date filter: 2013 to April 1, 2018 | 1888 |
| **HIC Search** | | | | |
| **Database searched** | **Date searched** | **Search Strategy** | **Filters** | **Results** |
| Pubmed | April 1, 2018 | (infant, newborn[mesh] OR infant[mesh] OR newborn* OR neonat* OR baby OR babies OR infant*) AND (ANDORRA OR ANTIGUA OR BARBUDA OR ARUBA OR AUSTRALIA OR AUSTRIA OR BAHAMAS OR BAHRAIN OR BARBADOS OR BELGIUM OR BERMUDA OR BRITISH VIRGIN ISLANDS OR bvi[tiab] OR BRUNEI OR CANADA OR CAYMAN ISLANDS OR CHANNEL ISLANDS OR CHILE OR CROATIA OR CURACAO OR CYPRUS OR CZECH REPUBLIC OR DENMARK OR ESTONIA OR FAROE ISLANDS OR FINLAND OR FRANCE OR FRENCH POLYNESIA OR GERMANY OR GIBRALTAR OR GREECE OR GREENLAND OR GUAM OR HONG KONG OR HUNGARY OR ICELAND OR IRELAND OR ISLE OF MAN OR ISRAEL OR ITALY OR JAPAN OR KOREA OR KUWAIT OR LATVIA OR LIECHTENSTEIN OR LITHUANIA OR LUXEMBOURG OR MACAO OR MALTA OR MONACO OR NETHERLANDS OR NEW CALEDONIA OR NEW ZEALAND OR NORTHERN MARIANA ISLANDS OR NORWAY OR OMAN OR PALAU OR PANAMA OR POLAND OR PORTUGAL OR PUERTO RICO OR QATAR OR SAN MARINO OR SAUDI ARABIA OR SEYCHELLES OR SINGAPORE OR SAINT MAARTEN OR SLOVAK REPUBLIC OR SLOVENIA OR SPAIN OR ST. KITTS OR NEVIS OR ST. MARTIN OR SWEDEN OR SWITZERLAND OR TRINIDAD OR TOBAGO OR TURKS AND CAICOS OR UNITED ARAB EMIRATES OR uae[tiab] OR UNITED KINGDOM OR uk[tiab] OR great britain OR london OR UNITED STATES OR us[tiab] OR URUGUAY OR VIRGIN ISLANDS OR vi[tiab] OR hic[tiab] OR "high income country" OR "high income countries" OR developed countries[mesh] OR Australia[mesh] OR Austria[mesh] OR Belgium[mesh] OR Canada[mesh] OR Chile[mesh] OR Czech Republic[mesh] OR Denmark[mesh] OR Estonia[mesh] OR Finland[mesh] OR France[mesh] OR Germany[mesh] OR Greece[mesh] OR Hungary[mesh] OR Iceland[mesh] OR Ireland[mesh] OR Israel[mesh] OR Italy[mesh] OR Japan[mesh] OR Korea[mesh] OR Latvia[mesh] OR Luxembourg[mesh] OR Mexico[mesh] OR Netherlands[mesh] OR New Zealand[mesh] OR Norway[mesh] OR Poland[mesh] OR Portugal[mesh] OR Slovak Republic[mesh] OR Slovenia[mesh] OR Spain[mesh] OR Sweden[mesh] OR Switzerland[mesh] OR Turkey[mesh] OR Great Britain[mesh] OR United States[mesh])  AND (randomized controlled trial[pt] OR rct[tiab] OR rcts[tiab] OR "randomized controlled trial" OR "randomized controlled trials" OR "randomized control trial" OR "Randomized control trials") | Date filter:  2013 to April 1, 2018 | 7382 |
