# Supplementary material for: Informed consent rates for neonatal randomized controlled trials in low- and lower middle-income versus high-income countries: A systematic review
Source: PLoS One. 2021 Mar 9;16(3):e0248263. doi: 10.1371/journal.pone.0248263 (PMC7943024; doi:10.1371/journal.pone.0248263)
Supplement: S2 Table — (DOCX) [file pone.0248263.s003.docx]

**S2 Table. Study characteristics among trials reporting a consent rate versus those that did not report a consent rate**

| **Study characteristics** | **Consent rate not reported (N=100)**  **n (%)** | **Consent rate reported (N=200)**  **n (%)** |  |
| --- | --- | --- | --- |
| **Setting** |  |  |  |
| LMIC | 58 (58.0) | 135 (67.5) |  |
| HIC | 42 (42.0) | 65 (32.5) |  |
| **Control type** |  |  |  |
| No placebo | 85 (85.0) | 159 (79.5) |  |
| Placebo | 15 (15.0) | 41 (20.5) |  |
| **Intervention** |  |  |  |
| Drug/nutrition | 55 (55.0) | 116 (58.0) |  |
| Medical device | 21 (21.0) | 41 (20.5) |  |
| Other | 24 (24.0) | 43 (21.5) |  |
| **Funding^a^** |  |  |  |
| Public | 11 (10.9) | 34 (17.0) |  |
| Private | 24 (23.8) | 21 (10.5) |  |
| Both | 8 (7.9) | 18 (9.0) |  |
| None | 21 (20.8) | 53 (26.5) |  |
| Not stated | 37 (36.6) | 74 (37.0) |  |
| **Timing of consent** |  |  |  |
| Antenatal | 12 (12.0) | 25 (12.5) |  |
| Postnatal | 81 (81.0) | 165 (82.5) |  |
| Both | 7 (7.0) | 10 (5.0) |  |
| **Publication year** |  |  |  |
| 2013 | 20 (20.0) | 39 (19.5) |  |
| 2014 | 14 (14.0) | 25 (13.0) |  |
| 2015 | 25 (25.0) | 49 (24.5) |  |
| 2016 | 23 (23.0) | 40 (20.0) |  |
| 2017 | 8 (8.0) | 33 (16.5) |  |
| 2018 | 10 (10.0) | 13 (6.5) |  |
| **Method of randomisation** |  |  |  |
| Individual | 100 (100.0) | 196 (98.0) |  |
| Cluster | 0 (0.0) | 3 (1.5) |  |
| Quasi | 0 (0.0) | 1 (0.5) |  |
| **Trial size** |  |  |  |
| Very large (n>1000) | 2 (2.0) | 6 (3.0) |  |
| Moderate to large (n=100–1000) | 45 (45.0) | 105 (52.5) |  |
| Small (n<100) | 53 (53.0) | 89 (44.5) |  |
| ^a^p=0.028  Note: All significant differences at the 95% confidence level are superscripted. A chi-squared test is used to derive the reported significant ‘funding’ p-value. | | | |
