# Supplementary material for: Informed consent rates for neonatal randomized controlled trials in low- and lower middle-income versus high-income countries: A systematic review
Source: PLoS One. 2021 Mar 9;16(3):e0248263. doi: 10.1371/journal.pone.0248263 (PMC7943024; doi:10.1371/journal.pone.0248263)
Supplement: S3 Table — (DOCX) [file pone.0248263.s004.docx]

**S3 Table. Low- and Lower Middle-Income Country Studies**

| **Study Title** | **First Author** | **Publication Year** | **Journal** | **Country**  **(ies)** | **Income Classification** | **Funding Classifi-cation** | **Method of Randomi-zation** | **Inter-**  **vention** | **Description of Control Arm** | **Timing of Consent** | **Number Screened** | **Number Enrolled** | **Consent Rate (%)** |
| --- | --- | --- | --- | --- | --- | --- | --- | --- | --- | --- | --- | --- | --- |
| T-piece or self inflating bag for positive pressure ventilation during delivery room resuscitation: An RCT | Thakur, A | 2015 | Resuscitation | India | Lower Middle-Income | None | Individual | Medical Device | No Placebo | Antenatal | 2146 | 90 | 100 |
| Gastric lavage for prevention of feeding problems in neonates with meconium-stained amniotic fluid: A randomised controlled trial | Sharma, P | 2014 | Pediatrics and International Child Health | India | Lower Middle-Income | Not Stated | Individual | Other | No Placebo | Antenatal | 782 | 538 | 95 |
| Effectiveness of 4% chlorhexidine umbilical cord care on neonatal mortality in Southern Province, Zambia (ZamCAT): a cluster-randomised controlled trial | Semrau, K | 2016 | Lancet Global Health | Zambia | Lower Middle-Income | Private | Cluster | Drug/  Nutrition | No Placebo | Antenatal | 42324 | 39679 | 97 |
| Comparison of three types of intervention to enhance placental redistribution in term newborns: Randomized control trial | Yadav, A | 2015 | Journal of Perinatology | India | Lower Middle-Income | Not Stated | Individual | Other | No Placebo | Antenatal | 1400 | 300 | 78 |
| Phenobarbitone in rh hemolytic disease of the newborn: A randomized double-blinded placebo-controlled trial | Venkatna-rayan, K | 2013 | Journal of Tropical Pediatrics | India | Lower Middle-Income | Not Stated | Individual | Drug/  Nutrition | No Placebo | Antenatal | 48 | 44 | 94 |
| Effect of Early versus Delayed Cord Clamping on Hematological Status of Preterm Infants at 6 wk of Age | Ranjit, T | 2015 | Indian Journal of Pediatrics | India | Lower Middle-Income | None | Individual | Other | No Placebo | Antenatal | 123 | 94 | 94 |
| Role of delay in umbilical cord clamping in reducing frequency of neonatal anaemia | Khalid, N | 2013 | Pakistan Journal of Medical and Health Sciences | Pakistan | Lower Middle-Income | Not Stated | Individual | Other | No Placebo | Antenatal | Not Stated | 109 | Un-available |
| Effect of therapeutic hypothermia on oxidative stress and outcome in term neonates with perinatal asphyxia: A randomized controlled trial | Joy, R | 2013 | Journal of Tropical Pediatrics | India | Lower Middle-Income | None | Individual | Medical Device | No Placebo | Antenatal | 160 | 116 | 97 |
| Comparison of two types of intervention to enhance placental redistribution in term infants: randomized control trial | Jaiswal, P | 2015 | European Journal of Pediatrics | India | Lower Middle-Income | Public | Individual | Other | No Placebo | Antenatal | 800 | 200 | 72 |
| Impact of early versus delayed cord clamping on mean hemoglobin level in term neonates | Ishaq, F | 2016 | Pakistan Paediatric Journal | Pakistan | Lower Middle-Income | Not Stated | Individual | Other | No Placebo | Antenatal | Not Stated | 200 | Un-available |
| Impact and feasibility of breast crawl in a tertiary care hospital | Girish, M | 2013 | Journal of Perinatology | India | Lower Middle-Income | Not Stated | Individual | Other | No Placebo | Antenatal | Not Stated | 100 | Un-available |
| The effect of BCG on iron metabolism in the early neonatal period: A controlled trial in Gambian neonates | Prenticea, S | 2015 | Vaccine | Gambia | Low-Income | Both Public and Private | Individual | Drug/  Nutrition | No Placebo | Antenatal | Not Stated | 120 | Un-available |
| Neonatal resuscitation using a laryngeal mask airway: A randomised trial in Uganda | Pejovic, N | 2018 | Archives of disease in Childhood | Uganda | Low-Income | Private | Individual | Medical Device | No Placebo | Antenatal | Not Stated | 49 | Un-available |
| Comparison between the efficacy of early and delayed umbilical cord clamping in preterm infants | Malik, AU | 2013 | Pakistan Journal of Medical and Health Sciences | Pakistan | Lower Middle-Income | Not Stated | Individual | Other | No Placebo | Antenatal | Not Stated | 80 | Un-available |
| Pulse oximeter sensor application during neonatal resuscitation: A randomized controlled trial | Louis, D | 2014 | Pediatrics | India | Lower Middle-Income | Public | Individual | Medical Device | No Placebo | Antenatal | 185 | 150 | 100 |
| Umbilical cord milking and hematological parameters in moderate to late preterm neonates: A randomized controlled trial | Kumar, B | 2015 | Indian Pediatrics | India | Lower Middle-Income | None | Individual | Other | No Placebo | Antenatal | 850 | 200 | 74 |
| Should delayed cord clamping be the No Placebo in term low risk deliveries? A randomized controlled trial from a medical college hospital in south India | Krishnan, L | 2015 | Journal of Clinical Neonatology | India | Lower Middle-Income | None | Individual | Other | No Placebo | Antenatal | 364 | 86 | 25 |
| Feasibility and safety of ALVAC-HIV vCP1521 vaccine in HIV-exposed infants in Uganda: results from the first HIV vaccine trial in infants in Africa | Kintu, K | 2013 | Journal of Acquired Immune Deficiency Syndromes | Uganda | Low-Income | Public | Individual | Drug/  Nutrition | Placebo | Antenatal | 222 | 60 | 100 |
| Effect of delayed cord clamping on hematocrit, and thermal and hemodynamic stability in preterm neonates: A randomized controlled trial | Dipak, N | 2017 | Indian Pediatrics | India | Lower Middle-Income | None | Individual | Other | No Placebo | Antenatal | 226 | 78 | 68 |
| Placental transfusion in preterm neonates of 30–33 weeks’ gestation: a randomized controlled trial | Das, B | 2018 | Journal of Perinatology | India | Lower Middle-Income | Not Stated | Individual | Other | No Placebo | Antenatal | 534 | 461 | 100 |
| Evaluation of compliance to congenital chagas disease treatment: Results of a randomised trial in Bolivia | Chippaux, JP | 2013 | Transactions of the Royal Society of Tropical Medicine and Hygiene | Bolivia | Lower Middle-Income | Both Public and Private | Individual | Drug/  Nutrition | No Placebo | Antenatal | 3579 | 124 | 99 |
| Newly born low birthweight infants stabilise better in skin-to-skin contact than when separated from their mothers: a randomised controlled trial | Chi Luong, K | 2016 | Acta Paediatrica | Vietnam | Lower Middle-Income | None | Individual | Other | No Placebo | Antenatal | 2230 | 100 | Un-available |
| Endotracheal suction for nonvigorous neonates born through meconium stained amniotic fluid: A randomized controlled trial | Chettri, S | 2015 | Journal of Pediatrics | India | Lower Middle-Income | Not Stated | Individual | Medical Device | No Placebo | Antenatal | 1271 | 122 | 100 |
| Endotracheal suction in term non vigorous Meconium stained neonates-A Pilot study | Nangiaa, S | 2016 | Resuscitation | India | Lower Middle-Income | Not Stated | Individual | Medical Device | No Placebo | Antenatal | 1176 | 175 | Un-available |
| Effect of 40-cm segment umbilical cord milking on hemoglobin and serum ferritin at 6 months of age in full-term infants of anemic and non-anemic mothers | Bora, R | 2015 | Journal of Perinatology | India | Lower Middle-Income | Not Stated | Individual | Other | No Placebo | Antenatal | 2712 | 200 | 96 |
| Nasal Jet-CPAP (variable flow) versus Bubble-CPAP in preterm infants with respiratory distress: An open label, randomized controlled trial | Bhatti, A | 2015 | Journal of Perinatology | India | Lower Middle-Income | Not Stated | Individual | Medical Device | No Placebo | Antenatal | 358 | 170 | 70 |
| Wrapping newborn infants in cloth and newspaper after delivery led to higher temperatures on arrival at the neonatal intensive care unit | Agrawal, N | 2017 | Acta Paediatrica | India | Lower Middle-Income | None | Individual | Medical Device | No Placebo | Antenatal | 100 | 100 | 100 |
| Effects of Delayed Umbilical Cord Clamping vs Early Clamping on Anemia in Infants at 8 and 12 Months: A Randomized Clinical Trial | KC, A | 2017 | JAMA Pediatrics | Nepal | Low-Income | Both Public and Private | Individual | Other | No Placebo | Antenatal | 2588 | 540 | 66 |
| A randomized controlled trial to evaluate the role of brief delay in cord clamping in preterm neonates (34-36 weeks) on shortterm neurobehavioural outcome | Datta, V | 2017 | Journal of Tropical Pediatrics | India | Lower Middle-Income | Not Stated | Individual | Other | No Placebo | Antenatal | 151 | 120 | 94 |
| Role of glycopyrrolate in healing of anastomotic dehiscence after primary repair of esophageal atresia in a low resource setting—A randomized controlled study | Vaghela, M | 2017 | Journal of Pediatric Surgery | India | Lower Middle-Income | None | Individual | Drug/  Nutrition | Placebo | Postnatal | 47 | 42 | 100 |
| Heparin for prolonging peripheral intravenous catheter use in neonates: A randomized controlled trial | Upadhyay, A | 2015 | Journal of Perinatology | India | Lower Middle-Income | Not Stated | Individual | Drug/  Nutrition | No Placebo | Postnatal | 130 | 120 | 92 |
| Routine versus intensive monitoring of serum bilirubin levels in neonatal jaundice | Ulman, S | 2015 | Perinatology | India | Lower Middle-Income | Not Stated | Individual | Other | No Placebo | Postnatal | Not Stated | 102 | Un-available |
| Effect of oral stimulation on feeding performance and weight gain in preterm neonates: a randomised controlled trial | Thakkar, P | 2018 | Paediatrics and International Child Health | India | Lower Middle-Income | Not Stated | Individual | Other | No Placebo | Postnatal | 130 | 102 | 96 |
| To evaluate and compare the efficacy of combined sucrose and non-nutritive sucking for analgesia in newborns undergoing minor painful procedure: A randomized controlled trial | Thakkar, P | 2016 | Journal of Perinatology | India | Lower Middle-Income | Not Stated | Individual | Drug/  Nutrition | No Placebo | Postnatal | 300 | 180 | 99 |
| Monotherapy with amikacin or piperacillin-tazobactum empirically in neonates at risk for early-onset sepsis: A randomized controlled trial | Tewari, V | 2014 | Journal of Tropical Pediatrics | India | Lower Middle-Income | Not Stated | Individual | Drug/  Nutrition | No Placebo | Postnatal | 204 | 187 | 98 |
| Early versus late enteral feeding in preterm intrauterine growth restricted neonates with antenatal doppler abnormalities: An open-label randomized trial | Tewari, V | 2018 | Journal of Tropical Pediatrics | India | Lower Middle-Income | Not Stated | Individual | Drug/  Nutrition | No Placebo | Postnatal | 77 | 62 | 93 |
| Bacillus clausii for Prevention of Late-onset Sepsis in Preterm Infants: A Randomized Controlled Trial | Tewari, V | 2015 | Journal of Tropical Pediatrics | India | Lower Middle-Income | Not Stated | Individual | Drug/  Nutrition | Placebo | Postnatal | 326 | 244 | 94 |
| A randomized double-blind controlled trial comparing two regimens of Vitamin D supplementation in preterm neonates | Tergestina, M | 2016 | Journal of Perinatology | India | Lower Middle-Income | Private | Individual | Drug/  Nutrition | No Placebo | Postnatal | 210 | 120 | 95 |
| Efficacy of modified Tochen’s formula for optimum endotracheal tube placement in low birth weight neonates: an RCT | Tatwavedi, D | 2018 | Journal of Perinatology | India | Lower Middle-Income | Not Stated | Individual | Other | No Placebo | Postnatal | 92 | 70 | 99 |
| Does therapeutic hypothermia reduce acute kidney injury among term neonates with perinatal asphyxia? - A randomized controlled trial | Tanigasalam, V | 2016 | Journal of Maternal-Fetal and Neonatal Medicine | India | Lower Middle-Income | Not Stated | Individual | Medical Device | No Placebo | Postnatal | 150 | 120 | 96 |
| Effect of feeding schedule on time to reach full feeds in ELBW and VLBW neonates: A randomized trial | Tali, S | 2016 | Perinatology | India | Lower Middle-Income | None | Individual | Drug/Nutrition | No Placebo | Postnatal | 215 | 120 | 89 |
| Bubble cpap versus ventilator cpap in preterm neonates with early onset respiratory distress - A randomized controlled trial | Tagare, A | 2013 | Journal of Tropical Pediatrics | India | Lower Middle-Income | Not Stated | Individual | Medical Device | No Placebo | Postnatal | 259 | 145 | 90 |
| Effect of kangaroo mother care on growth and morbidity pattern in low birth weight infants | Swarnkar, K | 2016 | Journal of Krishna Institute of Medical Sciences University | India | Lower Middle-Income | Not Stated | Quasi | Other | No Placebo | Postnatal | 82 | 60 | 88 |
| Fluid supplementation versus no fluid supplementation in late preterm and term neonates with asymptomatic polycythemia: A randomized controlled trial | Sundaram, M | 2016 | Indian Pediatrics | India | Lower Middle-Income | None | Individual | Drug/Nutrition | No Placebo | Postnatal | 121 | 55 | Un-available |
| Facilitated tucking on pain in pre-term newborns during neonatal intensive care: A single blinded randomized controlled cross-over pilot trial | Sundaram, B | 2013 | Journal of Pediatric Re-habilitation Medicine | India | Lower Middle-Income | Not Stated | Individual | Other | No Placebo | Postnatal | 32 | 24 | 100 |
| Effect of very early skin to skin contact on success at breastfeeding and preventing early hypothermia in neonates | Srivastava, S | 2014 | Indian Journal of Public Health | India | Lower Middle-Income | Not Stated | Individual | Other | No Placebo | Postnatal | Not Stated | 298 | Un-available |
| Evaluation of the uptake and impact of neonatal Vitamin A supplementation delivered through the Lady Health Worker programme on neonatal and infant morbidity and mortality in rural Pakistan: An effectiveness trial | Soofi, S | 2017 | Archives of Disease in Childhood | Pakistan | Lower Middle-Income | Both Public and Private | Cluster | Drug/  Nutrition | Placebo | Postnatal | 11808 | 11028 | 95 |
| Does topical lidocaine reduce the pain associated with the insertion of nasal continuous positive airway pressure prongs in preterm infants?: A randomized, controlled pilot trial | Soliman, H | 2016 | Clinical Journal of Pain | Egypt | Lower Middle-Income | Not Stated | Individual | Drug/  Nutrition | No Placebo | Postnatal | 82 | 60 | 78 |
| Comparative efficacy of phenobarbital, phenytoin and lorazepam for the treatment of neonatal seizures: A randomized Trial | Solanki, D | 2015 | Journal of Clinical Neonatology | India | Lower Middle-Income | None | Individual | Drug/  Nutrition | No Placebo | Postnatal | 121 | 106 | 100 |
| A randomized trial of phototherapy with filtered sunlight in african neonates | Slusher, T | 2015 | New England Journal of Medicine | Nigeria | Lower Middle-Income | Both Public and Private | Individual | Other | No Placebo | Postnatal | 1468 | 447 | Un-available |
| Role of probiotics VSL#3 in prevention of suspected sepsis in low birthweight infants in India: A randomised controlled trial | Sinha, A | 2015 | BMJ Open | India | Lower Middle-Income | Public | Individual | Drug/  Nutrition | Placebo | Postnatal | 5927 | 1340 | 85 |
| Breast feeding as analgesia in neonates: A randomized controlled trial | Singh, R | 2016 | Journal of Nepal Pediatric Society | India | Lower Middle-Income | None | Individual | Drug/  Nutrition | No Placebo | Postnatal | Not Stated | 60 | Un-available |
| Comparative efficacy and safety of caffeine and aminophylline for apnea of prematurity in preterm (≤34 weeks) neonates: A randomized controlled trial | Shivakumar, M | 2017 | Indian Pediatrics | India | Lower Middle-Income | Public | Individual | Drug/  Nutrition | No Placebo | Postnatal | 284 | 240 | 100 |
| Glycerin suppository for promoting feeding tolerance in preterm very low birthweight neonates: A randomized controlled trial | Shinde, S | 2014 | Indian Pediatrics | India | Lower Middle-Income | None | Individual | Drug/  Nutrition | No Placebo | Postnatal | 68 | 50 | 100 |
| High-intensity light-emitting diode vs fluorescent tubes for intensive phototherapy in neonates | Sherbiny, H | 2016 | Paediatrics and International Child Health | Egypt | Lower Middle-Income | None | Individual | Medical Device | No Placebo | Postnatal | 231 | 200 | 100 |
| The effect of kangaroo ward care in comparison with “intermediate intensive care” on the growth velocity in preterm infant with birth weight <1100 g: randomized control trial | Sharma, D | 2016 | European Journal of Pediatrics | India | Lower Middle-Income | None | Individual | Other | No Placebo | Postnatal | 202 | 141 | 100 |
| Mineral- and vitamin-enhanced micronutrient powder reduces stunting in full-term low-birth-weight infants receiving nutrition, health, and hygiene education: a 2 x 2 factorial, cluster-randomized trial in Bangladesh | Shafique, S | 2016 | American Journal of Clinical Nutrition | Bangladesh | Lower Middle-Income | Both Public and Private | Cluster | Drug/  Nutrition | No Placebo | Postnatal | 1295 | 467 | 97 |
| Conventional Versus Prolonged Infusion of Meropenem in Neonates With Gram-negative Late-onset Sepsis: A Randomized Controlled Trial | Shabaan, A | 2017 | Pediatric Infectious Disease Journal | Egypt | Lower Middle-Income | None | Individual | Drug/  Nutrition | No Placebo | Postnatal | 127 | 102 | 95 |
| Pentoxifylline Therapy for Late-Onset Sepsis in Preterm Infants: A Randomized Controlled Trial | Shabaan, A | 2015 | Pediatric Infectious Disease Journal | Egypt | Lower Middle-Income | None | Individual | Drug/  Nutrition | Placebo | Postnatal | 163 | 120 | 83 |
| Effect of Withholding Phenobarbitone Maintenance in Neonatal Seizures: A Randomized Controlled Trial | Saxena, P | 2016 | Indian Pediatrics | India | Lower Middle-Income | Public | Individual | Drug/  Nutrition | Placebo | Postnatal | 184 | 152 | 94 |
| Immunogenicity and safety of early vs delayed BCG vaccination in moderately preterm | Saroha, M | 2015 | Human Vaccines and Immunotherapeutics | India | Lower Middle-Income | Not Stated | Individual | Drug/  Nutrition | No Placebo | Postnatal | Not Stated | 180 | Un-available |
| Feasibility of exclusive enteral feeds from birth in VLBW infants >1200 g - An RCT | Sanghvi, K | 2013 | Acta Paediatrica | India | Lower Middle-Income | None | Individual | Drug/  Nutrition | No Placebo | Postnatal | 75 | 46 | 100 |
| Effect of emollient therapy on clinical outcomes in preterm neonates in Pakistan:A randomised controlled trial | Salam, R | 2015 | Archives of Disease in Childhood: Fetal and Neonatal Edition | Pakistan | Lower Middle-Income | Private | Individual | Drug/  Nutrition | No Placebo | Postnatal | 270 | 258 | 97 |
| Expressed breast milk vs 25% Dextrose in procedural pain in neonates: A double blind randomized controlled trial | Sahoo, J | 2013 | Indian Pediatrics | India | Lower Middle-Income | None | Individual | Drug/  Nutrition | Placebo | Postnatal | 440 | 210 | 83 |
| Intermittent versus continuous phototherapy for the treatment of neonatal non-hemolytic moderate hyperbilirubinemia in infants more than 34 weeks of gestational age: a randomized controlled trial | Sachdeva, M | 2015 | European Journal of Pediatrics | India | Lower Middle-Income | None | Individual | Medical Device | No Placebo | Postnatal | 137 | 75 | 93 |
| Digital palpation of endotracheal tube tip as a method of confirming endotracheal tube position in neonates: An open-label, three-armed randomized controlled trial | Saboo, A | 2013 | Paediatric Anesthesia | India | Lower Middle-Income | None | Individual | Medical Device | No Placebo | Postnatal | 95 | 57 | Un-available |
| Role of enteric supplementation of Probiotics on late-onset sepsis by Candida species in preterm low birth weight neonates: A randomized, double blind, placebo-controlled trial | Roy, A | 2014 | North American Journal of Medical Sciences | India | Lower Middle-Income | Not Stated | Individual | Drug/  Nutrition | Placebo | Postnatal | 341 | 112 | 72 |
| Comparison of alcohol versus dry cord care in terms of cord separation time | Zaheer, M | 2016 | Pakistan Paediatric Journal | Pakistan | Lower Middle-Income | Not Stated | Individual | Drug/  Nutrition | No Placebo | Postnatal | Not Stated | 70 | Un-available |
| Effects of term infant formulas containing high sn-2 palmitate with and without oligofructose on stool composition, stool characteristics, and bifidogenicity | Yao, M | 2014 | Journal of Pediatric Gastroenterology and Nutrition | Philip-pines | Lower Middle-Income | Private | Individual | Drug/  Nutrition | No Placebo | Postnatal | Not Stated | 375 | Un-available |
| Comparing the outcome of single versus multiple session laser photoablation of flat neovascularization in zone 1 Aggressive posterior retinopathy of prematurity: A prospective randomized study | Vinekar, A | 2015 | Retina | India | Lower Middle-Income | Not Stated | Individual | Medical Device | No Placebo | Postnatal | Not Stated | 29 | Un-available |
| Efficacy of expressed breast milk in reducing pain during ROP screening-a randomized controlled trial | Rosali, L | 2015 | Journal of Tropical Pediatrics | India | Lower Middle-Income | Not Stated | Individual | Drug/  Nutrition | No Placebo | Postnatal | Not Stated | 40 | Un-available |
| Seven versus 10 days antibiotic therapy for culture-proven neonatal sepsis: A randomised controlled trial | Rohatgi, S | 2017 | Journal of Paediatrics and Child Health | India | Lower Middle-Income | Not Stated | Individual | Drug/  Nutrition | No Placebo | Postnatal | 936 | 132 | 86 |
| Analgesic efficacy of Oral Dextrose and breast milk during nasopharyngeal suctioning of preterm infants on CPAP: A blinded randomized controlled trial | Rodrigues, L | 2017 | Journal of Tropical Pediatrics | India | Lower Middle-Income | Not Stated | Individual | Drug/  Nutrition | No Placebo | Postnatal | 51 | 40 | 98 |
| Oral dextrose for analgesia in neonates during nasogastric tube insertion: A randomised controlled trial | Ravishankar, A | 2014 | Journal of Paediatrics and Child Health | India | Lower Middle-Income | Public | Individual | Drug/  Nutrition | Placebo | Postnatal | Not Stated | 150 | Un-available |
| Comparison of two empiric antibiotic regimen in late onset neonatal sepsis-a randomized controlled trial | Ramasamy, S | 2014 | Journal of Tropical Pediatrics | India | Lower Middle-Income | Not Stated | Individual | Drug/  Nutrition | No Placebo | Postnatal | 135 | 90 | 95 |
| Effect of therapeutic hypothermia on myocardial dysfunction in term neonates with perinatal asphyxia – a randomized controlled trial | Rakesh, K | 2017 | Journal of Maternal-Fetal and Neontal Medicine | India | Lower Middle-Income | Public | Individual | Medical Device | No Placebo | Postnatal | 150 | 120 | 96 |
| Treating perinatal asphyxia with theophylline at birth helps to reduce the severity of renal dysfunction in term neonates | Raina, A | 2016 | Acta Paediatrica | India | Lower Middle-Income | None | Individual | Drug/  Nutrition | Placebo | Postnatal | Not Stated | 159 | Un-available |
| Volume guarantee ventilation in the weaning phase of preterm infants | Khashaba, M | 2015 | Egyptian Pediatric Association Gazette | Egypt | Lower Middle-Income | Not Stated | Individual | Medical Device | No Placebo | Postnatal | Not Stated | 40 | Un-available |
| Comparison of continuous with intermittent phototherapy in the treatment of neonatal jaundice | Khaliq, A | 2016 | Journal of Postgraduate Medical Institute | Pakistan | Lower Middle-Income | Not Stated | Individual | Medical Device | No Placebo | Postnatal | Not Stated | 258 | Un-available |
| A randomized controlled trial of burping for the prevention of colic and regurgitation in healthy infants | Kaur, R | 2015 | Child: Care, Health and Development | India | Lower Middle-Income | None | Individual | Other | No Placebo | Postnatal | 80 | 71 | 96 |
| Efficacy of bovine lactoferrin supplementation in preventing late-onset sepsis in low birth weight neonates: A randomized placebo-Controlled clinical trial | Kaur, G | 2015 | Journal of Tropical Pediatrics | India | Lower Middle-Income | Not Stated | Individual | Drug/  Nutrition | Placebo | Postnatal | 155 | 132 | 97 |
| Oral Dextrose for Pain Management during Laser Treatment of Retinopathy of Prematurity under Topical Anesthesia | Kataria, M | 2015 | Indian Journal of Pediatrics | India | Lower Middle-Income | None | Individual | Drug/  Nutrition | No Placebo | Postnatal | 26 | 24 | 100 |
| Effect of multisensory stimulation on neuromotor development in preterm infants | Kanagasabai, P | 2013 | Indian Journal of Pediatrics | India | Lower Middle-Income | None | Individual | Other | No Placebo | Postnatal | 57 | 50 | 100 |
| Early versus late enteral prophylactic iron supplementation in preterm very low birth weight infants: A randomised controlled trial | Joy, R | 2014 | Archives of Disease in Childhood: Fetal and Neonatal Edition | India | Lower Middle-Income | Public | Individual | Drug/  Nutrition | No Placebo | Postnatal | 166 | 104 | 90 |
| Comparison of non-synchronized nasal intermittent positive pressure ventilation versus nasal continuous positive airway pressure as post-extubation respiratory support in preterm infants with respiratory distress syndrome: A randomized controlled trial | Jasani, B | 2016 | Journal of Maternal-Fetal and Neonatal Medicine | India | Lower Middle-Income | Not Stated | Individual | Medical Device | No Placebo | Postnatal | 90 | 63 | 81 |
| Slow versus rapid enteral feed in preterm neonates with antenatal absent end diastolic flow | Jain, S | 2016 | Journal of Maternal-Fetal and Neonatal Medicine | India | Lower Middle-Income | Not Stated | Individual | Drug/  Nutrition | No Placebo | Postnatal | 159 | 83 | 100 |
| Probiotics for promoting feed tolerance in very low birth weight neonates — A randomized controlled trial | Shashidhar, A | 2017 | Indian Pediatrics | India | Lower Middle-Income | None | Individual | Drug/  Nutrition | No Placebo | Postnatal | 162 | 104 | 100 |
| Role of probiotics in prevention of nectrotizing enterocolitis in preterm low birth weight neonates | Hussain, M | 2016 | Pakistan Journal of Medical and Health Sciences | Pakistan | Lower Middle-Income | Not Stated | Individual | Drug/  Nutrition | No Placebo | Postnatal | Not Stated | 300 | Un-available |
| Short-term outcome of magnesium sulfate infusion in perinatal asphyxia | Hossain, M | 2013 | Mymensingh Medical Journal | Bangladesh | Lower Middle-Income | Not Stated | Individual | Drug/  Nutrition | Placebo | Postnatal | Not Stated | 50 | Un-available |
| Evaluation of phototherapy with reflectors: A randomized controlled trial | El Sayed Hashim, M | 2015 | International Journal of Pediatrics and Adolescent Medicine | Egypt | Lower Middle-Income | None | Individual | Medical Device | No Placebo | Postnatal | 99 | 65 | 88 |
| Nasal mask versus nasal prongs for delivering nasal continuous positive airway pressure in preterm infants with respiratory distress: A randomized controlled trial | Goel, S | 2015 | Indian Pediatrics | India | Lower Middle-Income | None | Individual | Medical Device | No Placebo | Postnatal | 181 | 118 | 83 |
| Role of prophylactic antibiotics in neonates born through meconium-stained amniotic fluid (MSAF)—a randomized controlled trial | Goel, A | 2015 | European Journal of Pediatrics | India | Lower Middle-Income | None | Individual | Drug/  Nutrition | No Placebo | Postnatal | 384 | 250 | 95 |
| The role of early inhaled budesonide therapy in meconium aspiration in term newborns: A randomized control study | Garg, N | 2016 | Journal of Maternal-Fetal and Neonatal Medicine | India | Lower Middle-Income | None | Individual | Drug/  Nutrition | Placebo | Postnatal | Not Stated | 78 | Un-available |
| Effect of therapeutic hypothermia on chromosomal aberration in perinatal asphyxia | Gane, B | 2016 | Journal of Pediatric Neuro-sciences | India | Lower Middle-Income | Public | Individual | Medical Device | No Placebo | Postnatal | Not Stated | 85 | Un-available |
| Effect of therapeutic hypothermia on DNA damage and neurodevelopmental outcome among term neonates with perinatal asphyxia: A randomized controlled trial | Gane, B | 2014 | Journal of Tropical Pediatrics | India | Lower Middle-Income | Public | Individual | Medical Device | No Placebo | Postnatal | 187 | 122 | 92 |
| Topical treatment of major omphalocoele: Acacia nilotica versus povidone-iodine: A randomised controlled study | Eltayeb, A | 2015 | African Journal of Paediatric Surgery | Egypt | Lower Middle-Income | None | Individual | Drug/  Nutrition | No Placebo | Postnatal | Not Stated | 24 | Un-available |
| Comparative Study of the Effects of Continuous Positive Airway Pressure and Nasal High-Flow Therapy on Diaphragmatic Dimensions in Preterm Infants | El-Mogy, M | 2018 | American Journal of Perinatology | Egypt | Lower Middle-Income | None | Individual | Medical Device | No Placebo | Postnatal | 38 | 24 | 85 |
| Comparative study of the efficacy and safety of paracetamol, ibuprofen, and indomethacin in closure of patent ductus arteriosus in preterm neonates | El-Mashad, A | 2017 | European Journal of Pediatrics | Egypt | Lower Middle-Income | None | Individual | Drug/  Nutrition | No Placebo | Postnatal | Not Stated | 300 | Un-available |
| Sustained versus intermittent lung inflation for resuscitation of preterm infants: a randomized controlled trial | El-Chimi, M | 2017 | Journal of Maternal-Fetal and Neontal Medicine | Egypt | Lower Middle-Income | Not Stated | Individual | Other | No Placebo | Postnatal | 202 | 202 | 100 |
| Single dose recombinant erythropoietin versus moderate hypothermia for neonatal hypoxic ischemic encephalopathy in low resource settings | Sami El Shimi, M | 2014 | Journal of Maternal-Fetal and Neonatal Medicine | Egypt | Lower Middle-Income | Not Stated | Individual | Drug/  Nutrition | No Placebo | Postnatal | Not Stated | 45 | Un-available |
| Post discharge formula fortification of maternal human milk of very low birth weight preterm infants: An introduction of a feeding protocol in a University Hospital | El Sakka, A | 2016 | Pediatric Reports | Egypt | Lower Middle-Income | None | Individual | Drug/  Nutrition | No Placebo | Postnatal | 80 | 59 | 89 |
| Oral sucrose for pain in neonates during echocardiography: A Randomized Controlled Trial | Potana, N | 2015 | Indian Pediatrics | India | Lower Middle-Income | None | Individual | Drug/  Nutrition | No Placebo | Postnatal | 157 | 104 | Un-available |
| Safety and immunogenicity of neonatal pneumococcal conjugate vaccination in Papua New Guinean children: a randomised controlled trial | Pomat, W | 2013 | PLOS One | Papua New Guinea | Lower Middle-Income | Private | Individual | Drug/  Nutrition | No Placebo | Postnatal | 448 | 312 | Un-available |
| Phenobarbitone versus phenytoin for treatment of neonatal seizures: An open-label randomized controlled trial | Pathak, G | 2013 | Indian Pediatrics | India | Lower Middle-Income | None | Individual | Drug/  Nutrition | No Placebo | Postnatal | 115 | 115 | 97 |
| Efficacy of early neonatal supplementation with vitamin A to reduce mortality in infancy in Haryana, India (Neovita): A randomised, double-blind, placebo-controlled trial | Mazumder, S | 2015 | The Lancet | India | Lower Middle-Income | Both Public and Private | Individual | Nutrition | Placebo | Postnatal | 55,443 | 44984 | 96 |
| A randomised controlled trial of flow driver and bubble continuous positive airway pressure in preterm infants in a resource-limited setting | Mazmanyan, P | 2015 | Archives of Disease in Childhood | Armenia | Lower Middle-Income | None | Individual | Medical Device | No Placebo | Postnatal | 142 | 125 | 97 |
| Assessment of adequacy of supplementation of vitamin D in very low birth weight preterm neonates: A randomized controlled trial | Mathur, N | 2016 | Journal of Tropical Pediatrics | India | Lower Middle-Income | Not Stated | Individual | Drug/  Nutrition | No Placebo | Postnatal | 82 | 50 | 98 |
| Evaluation of duration of antibiotic therapy in neonatal bacterial meningitis: A randomized controlled trial | Mathur, N | 2015 | Journal of Tropical Pediatrics | India | Lower Middle-Income | Not Stated | Individual | Drug/  Nutrition | No Placebo | Postnatal | 130 | 70 | 100 |
| Role of dexamethasone in neonatal meningitis: A randomized controlled trial | Mathur, N | 2013 | Indian Journal of Pediatrics | India | Lower Middle-Income | None | Individual | Drug/  Nutrition | No Placebo | Postnatal | 101 | 80 | 100 |
| Zinc supplementation in preterm neonates and neurological development: A randomized controlled trial | Mathur, N | 2015 | Indian Pediatrics | India | Lower Middle-Income | None | Individual | Drug/  Nutrition | No Placebo | Postnatal | 130 | 100 | 97 |
| Erythropoietin monotherapy in perinatal asphyxia with moderate to severe encephalopathy: A randomized placebo-controlled trial | Malla, R | 2017 | Journal of Perinatology | India | Lower Middle-Income | Not Stated | Individual | Drug/  Nutrition | Placebo | Postnatal | 293 | 100 | 91 |
| Effect of topical application of human breast milk versus 4% chlorhexidine versus dry cord care on bacterial colonization and clinical outcomes of umbilical cord in preterm newborns | Lyngdoh, D | 2018 | Journal of Clinical Neonatology | India | Lower Middle-Income | None | Individual | Drug/  Nutrition | No Placebo | Postnatal | Not Stated | 105 | Un-available |
| The Effect of Oral Polio Vaccine at Birth on Infant Mortality: A Randomized Trial | Lund, N | 2015 | Clinical Infectious Diseases | Guinea-Bissau | Low-Income | Both Public and Private | Individual | Drug/  Nutrition | No Placebo | Postnatal | 7073 | 7012 | 100 |
| Influence of maternal factors on the successful outcome of kangaroo mother care in low birth-weight infants: A randomized controlled trial | Lumbanraja, S | 2016 | Journal of Neonatal-Perinatal Medicine | Indo-nesia | Lower Middle-Income | Not Stated | Individual | Other | No Placebo | Postnatal | 90 | 40 | 95 |
| Can use of low-cost white reflecting curtains increase the efficacy of phototherapy? | Lahiri, S | 2016 | Journal of Clinical Neonatology | India | Lower Middle-Income | None | Individual | Medical Device | No Placebo | Postnatal | 500 | 102 | 98 |
| Dextrose boluses versus burette dextrose infusions in prevention of hypoglycemia among preterms admitted at Mulago Hospital: an open label randomized clinical trial | Kutamba, E | 2014 | African Health Sciences | Uganda | Low-Income | Not Stated | Individual | Drug/  Nutrition | No Placebo | Postnatal | 260 | 140 | 100 |
| Comparison of the efficacy of oral 25% glucose with oral 24% sucrose for pain relief during heel lance in preterm neonates: A double blind randomized controlled trial | Kumari, S | 2016 | Journal of Tropical Pediatrics | India | Lower Middle-Income | Not Stated | Individual | Drug/  Nutrition | No Placebo | Postnatal | 176 | 94 | 85 |
| Clofibrate as an Adjunct to Phototherapy for Unconjugated Hyperbilirubinemia in Term Neonates | Kumar, P | 2017 | Indian Journal of Pediatrics | India | Lower Middle-Income | None | Individual | Drug/  Nutrition | No Placebo | Postnatal | 95 | 90 | 97 |
| Prophylactic methylxanthines for preventing extubation failure in the preterm neonates with the gestational age of ≤30 weeks: A randomized controlled trial | Kumar, M | 2017 | Iranian Journal of Neonatology | India | Lower Middle-Income | Public | Individual | Drug/  Nutrition | Placebo | Postnatal | 185 | 156 | 100 |
| Prophylactic fluconazole in very low birth weight infants admitted to neonatal intensive care unit: Randomized controlled trial | Kirpal, H | 2016 | Journal of Maternal-Fetal and Neonatal Medicine | India | Lower Middle-Income | None | Individual | Drug/  Nutrition | Placebo | Postnatal | 84 | 80 | 98 |
| Comparison of Stool Colonization in Premature Infants by Three Dose Regimes of a Probiotic Combination: A Randomized Controlled Trial | Dutta, S | 2014 | American Journal of Perinatology | India | Lower Middle-Income | Private | Individual | Drug/  Nutrition | Placebo | Postnatal | Not Stated | 149 | Un-available |
| Umbilical venous catheter versus peripherally inserted central catheter in neonates: A randomized controlled trial | Dongara, A | 2017 | Journal of Tropical Pediatrics | India | Lower Middle-Income | Not Stated | Individual | Medical Device | No Placebo | Postnatal | 517 | 144 | 50 |
| Effect of Differential Enteral Protein on Growth and Neurodevelopment in Infants <1500 g: A Randomized Controlled Trial | Dogra, S | 2017 | Journal of Pediatric Gastroenterology and Nutrition | India | Lower Middle-Income | Not Stated | Individual | Drug/  Nutrition | No Placebo | Postnatal | 158 | 120 | 85 |
| Pre-exchange albumin administration in neonates with hyperbilirubinemia: A randomized controlled trial | Dash, N | 2015 | Indian Pediatrics | India | Lower Middle-Income | Not Stated | Individual | Drug/  Nutrition | Placebo | Postnatal | 126 | 50 | 96 |
| Neurological Outcome at 30 Months of Age after Mild Hypothermia via Selective Head Cooling in Term Neonates with Perinatal Asphyxia Using Low-Cost CoolCap: A Single-Center Randomized Control Pilot Trial in India | Das, S | 2017 | Journal of Pediatric Neurology | India | Lower Middle-Income | Not Stated | Individual | Other | No Placebo | Postnatal | 66 | 66 | 100 |
| Efficacy of probiotics versus placebo in the prevention of necrotizing enterocolitis in preterm very low birth weight infants: A double-blind randomized controlled trial | Chowdhury, T | 2016 | Journal of the College of Physicians and Surgeons Pakistan | Bangla-desh | Lower Middle-Income | Not Stated | Individual | Drug/  Nutrition | No Placebo | Postnatal | 163 | 102 | 74 |
| Evaluation of breastfeeding and 30% glucose solution as analgesic measures in indigenous African term neonates | Chiabi, A | 2016 | Journal of Clinical Neonatology | Came-roon | Lower Middle-Income | None | Individual | Drug/  Nutrition | No Placebo | Postnatal | Not Stated | 100 | Un-available |
| Nasal masks or binasal prongs for delivering continuous positive airway pressure in preterm neonates—a randomised trial | Chandrasek-aran, A | 2017 | European Journal of Pediatrics | India | Lower Middle-Income | None | Individual | Medical Device | No Placebo | Postnatal | 152 | 72 | 100 |
| Yakson touch and kinesthetic stimulation on development of high-risk neonates in neonatal intensive care units: A randomized controlled trial | *Parashar, P* | 2018 | Journal of Clinical Neonatology | India | Lower Middle-Income | Private | Individual | Other | No Placebo | Postnatal | 112 | 28 | Un-available |
| Effect of nasal continuous positive airway pressure on infants with meconium aspiration syndrome a randomized clinical trial | Pandita, A | 2018 | JAMA Pediatrics | India | Lower Middle-Income | Not Stated | Individual | Medical Device | No Placebo | Postnatal | 149 | 135 | 99 |
| Role of sucrose in reducing painful response to orogastric tube insertion in preterm neonates | Pandey, M | 2013 | Indian Journal of Pediatrics | India | Lower Middle-Income | None | Individual | Drug/  Nutrition | No Placebo | Postnatal | 186 | 120 | 94 |
| Effect of Breast-Feeding and Maternal Holding in Relieving Painful Responses in Full-Term Neonates: A Randomized Clinical Trial | Mahmoud Osman, A | 2015 | The Journal of Perinatal and Neonatal Nursing | Jordan | Lower Middle-Income | Not Stated | Individual | Drug/  Nutrition | No Placebo | Postnatal | Not Stated | 128 | 95 |
| Effect of early skin-to-skin contact following normal delivery on incidence of hypothermia in neonates more than 1800 g: randomized control trial | Nimbalkar, S | 2014 | Journal of Perinatology | India | Lower Middle-Income | Not Stated | Individual | Other | No Placebo | Postnatal | 232 | 100 | 96 |
| Kangaroo mother care in reducing pain in preterm neonates on heel prick | Nimbalkar, S | 2013 | Indian Journal of Pediatrics | India | Lower Middle-Income | None | Individual | Other | No Placebo | Postnatal | 69 | 50 | 83 |
| Effect of Zinc Supplementation on Early Outcome of Neonatal Sepsis - A Randomized Controlled Trial | Newton, B | 2016 | Indian Journal of Pediatrics | India | Lower Middle-Income | Public | Individual | Drug/  Nutrition | No Placebo | Postnatal | 134 | 88 | 91 |
| Topical anesthesia or oral dextrose for the relief of pain in screening for retinopathy of prematurity: A randomized controlled double-blinded trial | Nesargi, S | 2015 | Journal of Tropical Pediatrics | India | Lower Middle-Income | None | Individual | Drug/  Nutrition | No Placebo | Postnatal | 65 | 27 | 77 |
| Trial of daily vitamin D supplementation in preterm infants | Natarajan, C | 2014 | Pediatrics | India | Lower Middle-Income | Public | Individual | Drug/  Nutrition | No Placebo | Postnatal | 130 | 94 | 86 |
| Topical oil application and trans-epidermal water loss in preterm very low birth weight infants-a randomized trial | Nangia, S | 2015 | Journal of Tropical Pediatrics | India | Lower Middle-Income | Not Stated | Individual | Drug/  Nutrition | No Placebo | Postnatal | 106 | 74 | 95 |
| Synbiotics for decreasing incidence of necrotizing enterocolitis among preterm neonates - A randomized controlled trial | Nandhini, L | 2016 | Journal of Maternal-Fetal and Neontal Medicine | India | Lower Middle-Income | Not Stated | Individual | Drug/  Nutrition | No Placebo | Postnatal | 280 | 220 | 98 |
| Effect of kangaroo mother care Vs expressed breast milk administration on pain associated with removal of adhesive tape in very low birth weight neonates: A randomized controlled trial | Nanavati, R | 2013 | Indian Pediatrics | India | Lower Middle-Income | None | Individual | Other | No Placebo | Postnatal | 66 | 50 | 89 |
| Initiating nasal continuous positive airway pressure in preterm neonates at 5 cm as against 7 cm did not decrease the need for mechanical ventilation | Murki, S | 2016 | Acta Paediatrica | India | Lower Middle-Income | None | Individual | Medical Device | No Placebo | Postnatal | 1340 | 271 | 56 |
| High versus low-dose caffeine for apnea of prematurity: a randomized controlled trial | Mohammed, S | 2015 | European Journal of Pediatrics | Egypt | Lower Middle-Income | None | Individual | Drug/  Nutrition | No Placebo | Postnatal | 167 | 120 | 86 |
| Zinc supplementation fails to increase the immunogenicity of oral poliovirus vaccine: A randomized controlled trial | Habiba, M | 2015 | Vaccine | Pakistan | Lower Middle-Income | Public | Individual | Drug/  Nutrition | Placebo | Postnatal | 718 | 404 | 90 |
| Evaluation of efficacy of skin cleansing with chlorhexidine in prevention of neonatal nosocomial sepsis - A randomized controlled trial | Gupta, B | 2016 | Journal of Maternal-Fetal and Neonatal Medicine | India | Lower Middle-Income | None | Individual | Drug/  Nutrition | Placebo | Postnatal | 150 | 140 | 97 |
| Fluid supplementation in management of neonatal hyperbilirubinemia: a randomized controlled trial | Goyal, P | 2017 | Journal of Maternal-Fetal and Neonatal Medicine | India | Lower Middle-Income | None | Individual | Drug/  Nutrition | No Placebo | Postnatal | 206 | 150 | 99 |
| In resource limited areas complete enteral feed in stable very low birth weight infants (1000-1500 g) started within 24 h of life can improve nutritional outcome | Bora, R | 2017 | Journal of Maternal-Fetal and Neonatal Medicine | India | Lower Middle-Income | Not Stated | Individual | Drug/  Nutrition | No Placebo | Postnatal | 227 | 107 | 100 |
| Early BCG-Denmark and Neonatal Mortality Among Infants Weighing <2500 g: A Randomized Controlled Trial | Biering-Sørensen, S | 2017 | Clinical Infectious Diseases | Guinea-Bissau | Low-Income | Both Public and Private | Individual | Drug/  Nutrition | No Placebo | Postnatal | 4154 | 4133 | 100 |
| Effect of position of infant during phototherapy in management of hyperbilirubinemia in late preterm and term neonates: A randomized controlled trial | Bhethanabhotla, S | 2013 | Journal of Perinatology | India | Lower Middle-Income | Not Stated | Individual | Other | No Placebo | Postnatal | 238 | 100 | 71 |
| Efficacy and safety of polythene wrap in preventing hypothermia in preterm and low birth weight neonates during transport: A randomized controlled trial | Bhavsar, S | 2015 | Perinatology | India | Lower Middle-Income | Not Stated | Individual | Medical Device | No Placebo | Postnatal | 343 | 101 | 74 |
| Keeping babies warm: A non-inferiority trial of a conductive thermal mattress | Bhat, S | 2015 | Archives of Disease in Childhood Fetal and Neonatal Edition | India | Lower Middle-Income | Private | Individual | Medical Device | No Placebo | Postnatal | Not Stated | 160 | Un-available |
| Syndrome Evaluation System (SES) versus Blood Culture (BACTEC) in the Diagnosis and Management of Neonatal Sepsis - A Randomized Controlled Trial | Vishnu Bhat, B | 2016 | Indian Journal Pediatrics | India | Lower Middle-Income | Both Public and Private | Individual | Other | No Placebo | Postnatal | 452 | 385 | 97 |
| Efficacy of zinc supplementation on serum calprotectin, inflammatory cytokines and outcome in neonatal sepsis–a randomized controlled trial | Banupriya, N | 2016 | The Journal of Maternal-Fetal and Neonatal Medicine | India | Lower Middle-Income | Not Stated | Individual | Drug/  Nutrition | No Placebo | Postnatal | 183 | 134 | 92 |
| Short Term Oral Zinc Supplementation among Babies with Neonatal Sepsis for Reducing Mortality and Improving Outcome – A Double-Blind Randomized Controlled Trial | Banupriya, N | 2018 | Indian Journal Pediatrics | India | Lower Middle-Income | Public | Individual | Drug/  Nutrition | No Placebo | Postnatal | 203 | 150 | 92 |
| Effect of Sucrose Analgesia, for Repeated Painful Procedures, on Short-term Neurobehavioral Outcome of Preterm Neonates: A Randomized Controlled Trial | Banga, S | 2016 | Journal of Tropical Pediatrics | India | Lower Middle-Income | Not Stated | Individual | Drug/  Nutrition | Placebo | Postnatal | 152 | 106 | 96 |
| Effect of two different doses of parenteral amino acid supplementation on postnatal growth of very low birth weight neonates - A randomized controlled trial | Ruchinnana-vati, H | 2013 | Indian Pediatrics | India | Lower Middle-Income | None | Individual | Drug/  Nutrition | No Placebo | Postnatal | 408 | 150 | 72 |
| Bovine colostrum in prevention of necrotizing enterocolitis and sepsis in very low birth weight neonates: A randomized, double-blind, placebo-controlled pilot trial | Balachandr-an, B | 2017 | Journal of Tropical Pediatrics | India | Lower Middle-Income | Private | Individual | Drug/  Nutrition | Placebo | Postnatal | 3130 | 86 | 99 |
| Oromotor stimulation for transition from gavage to full oral feeding in preterm neonates: A Randomized controlled trial | Bala, P | 2016 | Indian Pediatrics | India | Lower Middle-Income | None | Individual | Other | No Placebo | Postnatal | Not Stated | 51 | Un-available |
| Efficacy, immunogenicity, and safety of two doses of a tetravalent rotavirus vaccine RRV-TV in Ghana with the first dose administered during the neonatal period | Armah, G | 2013 | Journal of Infectious Diseases | Ghana | Lower Middle-Income | Private | Individual | Drug/  Nutrition | No Placebo | Postnatal | 1029 | 998 | Un-available |
| Double Volume Exchange Transfusion in Severe Neonatal Sepsis | Somasekhara Aradhya, A | 2016 | Indian Journal of Pediatrics | India | Lower Middle-Income | None | Individual | Other | No Placebo | Postnatal | 621 | 83 | 98 |
| A comparative clinical study to evaluate the effect of punarnava mool churna and bhumyamalaki panchanga churna in physiological jaundice | Anju | 2014 | International Journal of Research in Ayurveda and Pharmacy | India | Lower Middle-Income | None | Individual | Drug/  Nutrition | No Placebo | Postnatal | Not Stated | 82 | Un-available |
| Role of gastric lavage in vigorous neonates born with meconium stained amniotic fluid | Ameta, G | 2013 | Indian Journal of Pediatrics | India | Lower Middle-Income | None | Individual | Medical Device | No Placebo | Postnatal | 361 | 244 | 98 |
| Does Positioning Affect Tracheal Aspiration of Gastric Content in Ventilated Infants? | Aly, H | 2015 | Gastroenterology | Egypt | Lower Middle-Income | Not Stated | Individual | Other | No Placebo | Postnatal | Not Stated | 34 | Un-available |
| Medically Graded Honey Supplementation Formula to Preterm infnats as a prebiotic: a randomized controlled trial | Aly, H | 2017 | Nutrition | Egypt | Lower Middle-Income | Not Stated | Individual | Drug/  Nutrition | No Placebo | Postnatal | Not Stated | 40 | Un-available |
| Melatonin use for neuroprotection in perinatal asphyxia: a randomized controlled pilot study | Aly, H | 2015 | Journal of Perinatology | Egypt | Lower Middle-Income | Not Stated | Individual | Drug/  Nutrition | No Placebo | Postnatal | Not Stated | 30 | Un-available |
| Oral paracetamol versus oral ibuprofen for treatment of patent ductus arteriosus | Al-lawama, M | 2017 | Journal of International Medical Research | Jordan | Lower Middle-Income | Public | Individual | Drug/  Nutrition | No Placebo | Postnatal | 128 | 22 | 92 |
| A Randomized Trial Comparing Efficacy of Bubble and Ventilator Derived Nasal CPAP in Very Low Birth Weight Neonates with Respiratory Distres | Agarwal, S | 2016 | Journal of Clinical and Diagnostic Research | India | Lower Middle-Income | Not Stated | Individual | Medical Device | No Placebo | Postnatal | 96 | 68 | Un-available |
| Randomized control trial of Kangaroo Mother Care in low birth weight babies at a tertiary level hospital | Acharya, N | 2014 | Journal of Nepal Pediatric Society | Nepal | Low-Income | None | Individual | Other | No Placebo | Postnatal | 385 | 126 | Un-available |
| Oral ibuprofen in full-term neonate’s patent ductus arteriosus closure: Discerning the role of prostaglandin, vascular endothelial growth factor, and immature platelet fraction | Yantie, N | 2017 | Current Pediatric Research | Indonesia | Lower Middle-Income | Not Stated | Individual | Drug/  Nutrition | Placebo | Postnatal | 135 | 64 | 99 |
| Effect of oil massage on growth in preterm neonates less than 1800 g: A randomized control trial | Kumar, J | 2013 | Indian Journal of Pediatrics | India | Lower Middle-Income | None | Individual | Other | No Placebo | Postnatal | 54 | 52 | 100 |
| Zinc supplementation for neonatal hyperbilirubinemia: A randomized controlled trial | Kumar, A | 2014 | Indian Pediatrics | India | Lower Middle-Income | Private | Individual | Drug/  Nutrition | Placebo | Postnatal | 284 | 80 | 86 |
| Dextrose-containing intraoperative fluid in neonates: A randomized controlled trial | Datta, P | 2016 | Paediatric Anaesthesia | India | Lower Middle-Income | Public | Individual | Drug/  Nutrition | No Placebo | Postnatal | 52 | 45 | 100 |
| Enteral paracetamol or intravenous indomethacin for closure of patent ductus arteriosus in preterm neonates: A randomized controlled trial | Dash, S | 2015 | Indian Pediatrics | India | Lower Middle-Income | None | Individual | Drug/  Nutrition | Placebo | Postnatal | 171 | 77 | 76 |
| Assessment of neonatal pain during heel prick: Lancet vs needle-A randomized controlled study | Britto, C | 2017 | Journal of Tropical Pediatrics | India | Lower Middle-Income | Public | Individual | Medical Device | No Placebo | Postnatal | 48 | 40 | 93 |
| Selenium supplementation for prevention of late-onset sepsis in very low birth weight preterm neonates | Aggarwal, R | 2016 | Journal of Tropical Pediatrics | India | Lower Middle-Income | Not Stated | Individual | Drug/  Nutrition | Placebo | Postnatal | 136 | 114 | 97 |
| Born not breathing: A randomised trial comparing two self-inflating bag-masks during newborn resuscitation in Tanzania | Thallinger, M | 2017 | Resuscitation | Tanzania | Low-Income | Both Public and Private | Individual | Medical Device | No Placebo | Both Antenatal and Prenatal | 6110 | 349 | 99 |
| Immunogenicity of a new routine vaccination schedule for global poliomyelitis prevention: An open-label, randomised controlled trial | Sutter, R | 2015 | The Lancet | India | Lower Middle-Income | Public | Individual | Drug/  Nutrition | No Placebo | Both Antenatal and Prenatal | Not Stated | 900 | Un-available |
| Immunogenicity of Different Routine Poliovirus Vaccination Schedules: A Randomized, Controlled Trial in Karachi, Pakistan | Saleem, A | 2018 | Journal of Infectious Diseases | Pakistan | Lower Middle-Income | Public | Individual | Drug/  Nutrition | No Placebo | Both Antenatal and Prenatal | 1481 | 900 | 88 |
| Hepatitis B vaccination with or without hepatitis B immunoglobulin at birth to babies born of HBsAg-positive mothers prevents overt HBV transmission but may not prevent occult HBV infection in babies: A randomized controlled trial | Pande, C | 2013 | Journal of Viral Hepatitis | India | Lower Middle-Income | Public | Individual | Drug/  Nutrition | Placebo | Both Antenatal and Prenatal | 283 | 259 | 96 |
| Early rescue Neopuff for infants with transient tachypnea of newborn: a randomized controlled trial | Mahmoud Osman, A | 2017 | Journal of Maternal-Fetal and Neontal Medicine | Egypt | Lower Middle-Income | Not Stated | Individual | Drug/  Nutrition | No Placebo | Both Antenatal and Prenatal | 100 | 78 | Un-available |
| High-Flow Nasal Cannula versus Nasal Continuous Positive Airway Pressure for Primary Respiratory Support in Preterm Infants with Respiratory Distress: A Randomized Controlled Trial | Murkia, S | 2018 | Neonatology | India | Lower Middle-Income | None | Individual | Medical Device | No Placebo | Both Antenatal and Prenatal | 1716 | 272 | 81 |
| Monovalent type-1 oral poliovirus vaccine given at short intervals in Pakistan: a randomised controlled, four-arm, open-label, non-inferiority trial | Mir, F | 2015 | Lancet Infectious Disease | Pakistan | Lower Middle-Income | Public | Individual | Drug/  Nutrition | No Placebo | Both Antenatal and Prenatal | 1905 | 829 | 72 |
| Human neonatal rotavirus vaccine (RV3-BB) to target rotavirus from birth | Bines, J | 2018 | The New England Journal of Medicine | Indo-nesia | Lower Middle-Income | Both Public and Private | Individual | Drug/  Nutrition | Placebo | Both Antenatal and Prenatal | Not Stated | 1649 | Un-available |
| Randomized trial of plastic bags to prevent term neonatal hypothermia in a resource-poor setting | Belsches, T | 2013 | Pediatrics | Zambia | Lower Middle-Income | Public | Individual | Medical Device | No Placebo | Both Antenatal and Prenatal | Not Stated | 275 | Un-available |
| Role of haridradi tailam application in prevention of nabhi paka (umbilical sepsis) in neonates | Raut, D | 2013 | International Journal of Research in Ayurveda and Pharmacy | India | Lower Middle-Income | Not Stated | Individual | Drug/  Nutrition | No Placebo | Postnatal | Not Stated | 60 | Un-available |
| Effect of topical application of chlorhexidine for umbilical cord care in comparison with conventional dry cord care on the risk of neonatal sepsis: A randomized controlled trial | Gathwala, G | 2013 | Journal of Tropical Pediatrics | India | Lower Middle-Income | None | Individual | Drug/  Nutrition | No Placebo | Postnatal | 146 | 140 | Un-available |
| Nebulized hypertonic saline to prevent ventilator associated pneumonia in premature infants, a randomized trial | Ezzeldin, Z | 2018 | The Journal of Maternal-Fetal and Neonatal Medicine | Egypt | Lower Middle-Income | None | Individual | Drug/  Nutrition | No Placebo | Postnatal | 120 | 100 | Un-available |
| The Efficacy of 1% Chloramphenicol Eye Ointment Versus 2.5% Povidone-Iodine Ophthalmic Solution in Reducing Bacterial Colony in Newborn Conjunctivae | Bramantyo, T | 2015 | Asia-Pacific Journal of Ophthal-mology | Indo-nesia | Lower Middle-Income | Not Stated | Individual | Drug/  Nutrition | No Placebo | Postnatal | Not Stated | 60 | Un-available |
| Reduction of neonatal pain following administration of 25% lingual dextrose: A randomized control trial | Nimbalkar, S | 2013 | Journal of Tropical Pediatrics | India | Lower Middle-Income | Not Stated | Individual | Drug/  Nutrition | No Placebo | Postnatal | Not Stated | 104 | Un-available |
| A comparative study to assess the effect of oil massage vs kangaroo mother care on changes in the physiological and neurobehavioural parameters among low birth weight babies | Biswal, A | 2015 | Indian Journal of Public Health Research and Development | India | Lower Middle-Income | None | Individual | Drug/  Nutrition | No Placebo | Both Antenatal and Prenatal | Not Stated | 160 | Un-available |
| Clinical outcomes of snuggle up position using positioning AIDS for preterm (27-32 weeks) infants | Sathish, Y | 2017 | Iranian Journal of Neonatology | India | Lower Middle-Income | Not Stated | Individual | Other | No Placebo | Postnatal | 72 | 56 | Un-available |
| Early versus late clamping of the umbilical cord in full-term neonates | Malhi, K | 2015 | Pakistan Journal of Medical and Health Sciences | Pakistan | Lower Middle-Income | Not Stated | Individual | Other | No Placebo | Antenatal | Not Stated | 200 | Un-available |
| Comparison of umbilical cord cleansing using sterile water and povidine iodine-spirit during early neonatal period: A double blind randomized control trial | Chawla, G | 2015 | Journal of Clinical and Diagnostic Research | India | Lower Middle-Income | Not Stated | Individual | Drug/  Nutrition | Placebo | Both Antenatal and Prenatal | Not Stated | 958 | Un-available |
| Randomised controlled trial of diazoxide for small for gestational age neonates with hyperinsulinaemic hypoglycaemia provided early hypoglycaemic control without adverse effects | Balachan-dran, B | 2018 | Acta Paediatrica | India | Lower Middle-Income | Public | Individual | Drug/  Nutrition | Placebo | Both Antenatal and Prenatal | 490 | 30 | 95 |
