# Supplementary material for: Informed consent rates for neonatal randomized controlled trials in low- and lower middle-income versus high-income countries: A systematic review
Source: PLoS One. 2021 Mar 9;16(3):e0248263. doi: 10.1371/journal.pone.0248263 (PMC7943024; doi:10.1371/journal.pone.0248263)
Supplement: S4 Table — (DOCX) [file pone.0248263.s005.docx]

**S4 Table. High-Income Country Studies**

| **Study Title** | **First Author** | **Publi-cation Year** | **Journal** | **Country**  **(ies)** | **Income Classifi-cation** | **Funding Classifi-cation** | **Method of Randomi-zation** | **Inter-vention** | **Description of Control Arm** | **Timing of Consent** | **Number Screened** | **Number Enrolled** | **Consent Rate %** |
| --- | --- | --- | --- | --- | --- | --- | --- | --- | --- | --- | --- | --- | --- |
| Supporting Play Exploration and Early Development Intervention From NICU to Home: A Feasibility Study | Dusing, S | 2015 | Pediatric Physical Therapy | USA | High-Income Country | Public | Individual | Other | No Placebo | Postnatal | Not Stated | 10 | 72 |
| Oral Sucrose for Heel Lance Increases Adenosine Triphosphate Use and Oxidative Stress in Preterm Neonates | Asmerom, Y | 2013 | Journal of Pediatrics | USA | High-Income Country | Public | Individual | Drug/  Nutrition | Placebo | Postnatal | Not Stated | 131 | 66 |
| Early versus delayed umbilical cord clamping in infants with congenital heart disease: a pilot, randomized, controlled trial | Backes, CH | 2015 | Journal of Perinatology | USA | High-Income Country | Private | Individual | Other | No Placebo | Antenatal | 188 | 30 | 70 |
| Timing of umbilical cord clamping among infants born at 22 through 27 weeks' gestation | Backes, CH | 2016 | Journal of Perinatology | USA | High-Income Country | Private | Individual | Other | No Placebo | Antenatal | 362 | 40 | 86 |
| Darbepoetin administration to neonates undergoing cooling for encephalopathy: a safety and pharmacokinetic trial | Baserga, M | 2015 | Pediatric Research | USA | High-Income Country | Private | Individual | Drug/  Nutrition | Placebo | Postnatal | 62 | 30 | 68 |
| A randomized trial of platelet transfusions over 30 vs 120 minutes: is there an effect on post-transfusion platelet counts? | Dannaway, DC | 2013 | Journal of Perinatology | USA | High-Income Country | Public | Individual | Drug/  Nutrition | No Placebo | Postnatal | Not Stated | 22 | 79 |
| A Pilot Randomized Controlled Trial of Early versus Routine Caffeine in Extremely Premature Infants | Katheria, A | 2015 | Am J Perinatol | USA | High-Income Country | Private | Individual | Drug/  Nutrition | No Placebo | Both Antenatal and Prenatal | 63 | 21 | 100 |
| Safety and pharmacokinetics of multiple dose myo-inositol in preterm infants | Phelps, D | 2016 | Pediatric Research | USA | High-Income Country | Public | Individual | Drug/  Nutrition | Placebo | Postnatal | 636 | 126 | 38 |
| Effects of Placental Transfusion on Neonatal and 18 Month Outcomes in Preterm Infants: A Randomized Controlled Trial | Mercer, J | 2016 | J Pediatr | USA | High-Income Country | Both Public and Private | Individual | Other | No Placebo | Antenatal | 1139 | 582 | 70 |
| Treating EEG Seizures in Hypoxic Ischemic Encephalopathy: A Randomized Controlled Trial | Srinivasa-kumar, P | 2015 | Pediatrics | USA | High-Income Country | Private | Individual | Drug/  Nutrition | No Placebo | Postnatal | 91 | 69 | 97 |
| A comparison of 2 intravenous lipid emulsions: interim analysis of a randomized controlled trial | Nehra, D | 2014 | JPEN | USA | High-Income Country | Not Stated | Individual | Drug/  Nutrition | No Placebo | Postnatal | 26 | 19 | 86 |
| Influence of holding practice on preterm infant development | Neu, M | 2013 | MCN Am J Matern Child Nurs | USA | High-Income Country | Not Stated | Individual | Other | No Placebo | Postnatal | ?? | 87 | 40 |
| A comparative effectiveness study of continuous positive airway pressure-related skin breakdown when using different nasal interfaces in the extremely low birth weight neonate | Newnam, K | 2015 | Applied Nursing Research | USA | High-Income Country | None | Individual | Medical Device | No Placebo | Postnatal | 377 | 78 | 98 |
| A Pilot Study of Antithrombin Replacement Prior to Cardiopulmonary Bypass in Neonates | Niebler, R | 2015 | Artificial Organs | USA | High-Income Country | Both Public and Private | Individual | Drug/  Nutrition | Placebo | Postnatal | 34 | 8 | 28 |
| Effect of decreased parenteral soybean lipid emulsion on hepatic function in infants at risk for parenteral nutrition-associated liver disease: A pilot study | Rollins, M | 2013 | Journal of Pediatric Surgery | USA | High-Income Country | Both Public and Private | Individual | Drug/  Nutrition | No Placebo | Postnatal | ?? | 30 | 47 |
| The impact of neurally adjusted ventilatory assist mode on respiratory severity score and energy expenditure in infants: a randomized crossover trial | Rosterman, J | 2018 | Journal of Perinatology | USA | High-Income Country | Both Public and Private | Individual | Medical Device | No Placebo | Postnatal | 149 | 24 | 69 |
| A Randomized-Controlled Trial Pilot Study Examining the Neurodevelopmental Effects of a 5-Week M Technique Intervention on Very Preterm Infants | Smith, J | 2014 | Advances in Neonatal Care | USA | High-Income Country | Private | Individual | Other | No Placebo | Postnatal | 45 | 20 | 95 |
| Randomised controlled trial of early frenotomy in breastfed infants with mild-moderate tongue-tie | Emond, A | 2014 | Arch Dis Child Fetal Neontal | UK | High-Income Country | Public | Individual | Other | No Placebo | Postnatal | 536 | 107 | 53 |
| Hypercapnia and acidosis during open and thoracoscopic repair of congenital diaphragmatic hernia and esophageal atresia: results of a pilot randomized controlled trial | Bishay, M | 2013 | Annals of Surgery | UK | High-Income Country | Private | Individual | Other | No Placebo | Postnatal | 36 | 20 | 83 |
| Olive Oil, Sunflower Oil or no Oil for Baby Dry Skin or Massage: A Pilot, Assessor-blinded, Randomized Controlled Trial (the Oil in Baby SkincaRE [OBSeRvE] Study) | Cooke, A | 2016 | Acta  Dermato-Venereologica | UK | High-Income Country | Public | Individual | Drug/  Nutrition | No Placebo | Postnatal | 4085 | 115 | 27 |
| Nutritional Evaluation and Optimisation in Neonates: a randomized, double-blind controlled trial of amino acid regimen and intravenous lipid composition in preterm parenteral nutrition | Uthaya, S | 2016 | Am J Clin Nutr | UK | High-Income Country | Public | Individual | Drug/  Nutrition | No Placebo | Both Ante-natal and Prenatal | 460 | 168 | 70 |
| Randomized, Placebo-Controlled Trial of Dobutamine for Low Superior Vena Cava Flow in Infants | Bravo, M | 2015 | Journal of Pediatrics | Spain | High-Income Country | Not Stated | Individual | Drug/  Nutrition | Placebo | Postnatal | 193 | 127 | 83 |
| Analgesia with breastfeeding in addition to skin-to-skin contact during heel prick | Gabriel, M | 2013 | Arch Dis Child Fetal Neontal | Spain | High-Income Country | Not Stated | Individual | Drug/  Nutrition | No Placebo | Postnatal | 156 | 136 | 89 |
| Humidified High Flow Nasal Cannula versus Nasal Continuous Positive Airway Pressure as an Initial Respiratory Support in Preterm Infants with Respiratory Distress: a Randomized, Controlled Non-Inferiority Trial | Shin, J | 2017 | Journal of Korean medical science | South Korea | High-Income Country | Not Stated | Individual | Medical Device | No Placebo | Postnatal | 237 | 87 | 81 |
| Improved Visual Perception in Very Low Birth Weight Infants on Enhanced Nutrient Supply | Blakstada, E | 2015 | Neonatology | Norway | High-Income Country | Not Stated | Individual | Drug/  Nutrition | No Placebo | Postnatal | 57 | 50 | 98 |
| Randomized controlled trial of oxygen saturation targets in very preterm infants: two year outcomes | Darlow, B | 2014 | J Pediatr | New Zealand | High-Income Country | Both Public and Private | Individual | Other | No Placebo | Postnatal | 587 | 340 | 85 |
| Vitamin D supplementation during pregnancy and infancy reduces aeroallergen sensitization: a randomized controlled trial | Grant, C | 2016 | Allergy | New Zealand | High-Income Country | Not Stated | Individual | Drug/  Nutrition | Placebo | Antenatal | 404 | 260 | 75 |
| Respiratory syncytial virus and recurrent wheeze in healthy preterm infants | Blanken, M | 2013 | New England Journal of Medicine | Nether-lands | High-Income Country | Both Public and Private | Individual | Drug/  Nutrition | Placebo | Postnatal | 1550 | 429 | 37 |
| The Bilirubin Albumin Ratio in the Management of Hyperbilirubinemia in Preterm Infants to Improve Neurodevelopmental Outcome: A Randomized Controlled Trial - BARTrial | Hulzebos, C | 2014 | Plos One | Nether-lands | High-Income Country | Public | Individual | Other | No Placebo | Postnatal | 934 | 615 | 84 |
| Effect of non-human neutral and acidic oligosaccharides on allergic and infectious diseases in preterm infants | Niele, N | 2013 | Eur J Pediatr | Nether-lands | High-Income Country | Private | Individual | Drug/  Nutrition | Placebo | Postnatal | 208 | 114 | 72 |
| Growth and fatty acid profiles of VLBW infants receiving a multicomponent lipid emulsion from birth | Vlaardinger-broek, H | 2014 | JPGN | Nether-lands | High-Income Country | Not Stated | Individual | Drug/  Nutrition | No Placebo | Postnatal | 367 | 98 | 47 |
| Treatment of patent ductus arteriosus (PDA) using ibuprofen: renal side-effects in VLBW and ELBW newborns | Bagnoli, F | 2013 | The Journal of Maternal-Fetal & Neonatal Medicine | Italy | High-Income Country | Not Stated | Individual | Drug/  Nutrition | Placebo | Postnatal | Not Stated | 134 | 93 |
| Higher docosahexaenoic acid, lower arachidonic acid and reduced lipid tolerance with high doses of a lipid emulsion containing 15% fish oil: a randomized clinical trial | D'Ascenzo, R | 2014 | Clin Nutr | Italy | High-Income Country | None | Individual | Drug/  Nutrition | No Placebo | Postnatal | 92 | 80 | 95 |
| Lutein and Zeaxanthin Supplementation in Preterm Very Low-Birth-Weight Neonates in Neonatal Intensive Care Units: A Multicenter Randomized Controlled Trial | Manzoni, P | 2013 | American Journal of Perinatology | Italy | High-Income Country | Private | Individual | Drug/  Nutrition | Placebo | Postnatal | 247 | 229 | 94 |
| The Effect of Optimally Timed Osteopathic Manipulative Treatment on Length of Hospital Stay in Moderate and Late Preterm Infants: Results from a RCT | Pizzolorusso, G | 2014 | Evidence-Based Complementary and Alternative Medicine | Italy | High-Income Country | Not Stated | Individual | Other | No Placebo | Postnatal | 209 | 110 | 100 |
| Continuous infusion of ibuprofen for treatment of patent ductus arteriosus in very low birth weight infants | Lago, P | 2013 | Neonatology | Italy | High-Income Country | Not Stated | Individual | Drug/  Nutrition | No Placebo | Postnatal | 308 | 112 | 97 |
| The effect of 5 intravenous lipid emulsions on plasma phytosterols in preterm infants receiving parenteral nutrition: a randomized clinical trial | Savini, S | 2013 | American Society for Nutrition | Italy | High-Income Country | None | Individual | Drug/  Nutrition | No Placebo | Postnatal | 205 | 150 | 94 |
| Preventive effects of oral probiotic on infantile colic: a prospective, randomised, blinded, controlled trial using Lactobacillus reuteri DSM 17938 | Savino, F | 2015 | Beneficial Microbes | Italy | High-Income Country | Private | Individual | Drug/  Nutrition | No Placebo | Postnatal | 138 | 113 | 88 |
| Lactobacillus paracasei subsp. paracasei F19 in Bell's stage 2 of necrotizing enterocolitis | Zampieri, N | 2013 | Minerva Paediatr | Italy | High-Income Country | Private | Individual | Drug/  Nutrition | No Placebo | Postnatal | 194 | 32 | 100 |
| Proactive enteral nutrition in moderately preterm small for gestational age infants: a randomized clinical trial | Zecca, E | 2014 | J Pediatr | Italy | High-Income Country | Not Stated | Individual | Drug/  Nutrition | No Placebo | Postnatal | 111 | 72 | 88 |
| A randomized pilot study comparing heated humidified high-flow nasal cannulae with NIPPV for RDS | Kugelman, A | 2015 | Pediatric Pulmonology | Israel | High-Income Country | Not Stated | Individual | Medical Device | No Placebo | Postnatal | 217 | 76 | 97 |
| High flow nasal cannula versus NCPAP, duration to full oral feeds in preterm infants: a randomised controlled trial | Glackin, S | 2017 | Arch Dis Child Fetal Neontal | Ireland | High-Income Country | Not Stated | Individual | Medical Device | No Placebo | Postnatal | 149 | 44 | 86 |
| 2% chlorhexidine-70% isopropyl alcohol versus 10% povidone-iodine for insertion site cleaning before central line insertion in preterm infants: a randomised trial | Kieran, E | 2018 | Arch Dis Child Fetal Neontal | Ireland | High-Income Country | Private | Individual | Drug/  Nutrition | No Placebo | Postnatal | 434 | 310 | 91 |
| The Effect of Upper Limb Massage on Infants' Venipuncture Pain | Chik, Y | 2017 | Pain Management Nursing | Hong Kong | High-Income Country | Not Stated | Individual | Other | No Placebo | Postnatal | Not Stated | 80 | 91 |
| Oral L-arginine supplementation and faecal calprotectin levels in very low birth weight neonates | Polycarpou, E | 2013 | Journal of Perinatology | Greece | High-Income Country | None | Individual | Drug/  Nutrition | Placebo | Postnatal | 171 | 83 | 72 |
| Closed-loop automatic oxygen control (CLAC) in preterm infants: a randomized controlled trial | Hallen-berger, A | 2014 | Pediatrics | Germany | High-Income Country | Private | Individual | Medical Device | No Placebo | Postnatal | 52 | 44 | 96 |
| Effects of standardized acoustic stimulation in premature infants: a randomized controlled trial | Wirth, L | 2016 | Journal of Perinatology | Germany | High-Income Country | Not Stated | Individual | Other | No Placebo | Postnatal | 108 | 62 | 82 |
| Dose-Finding Study of Omeprazole on Gastric pH in Neonates with Gastro-Esophageal Acid Reflux Using a Bayesian Sequential Approach | Kaguelidou, F | 2016 | Plos ONE | France | High-Income Country | Public | Individual | Drug/  Nutrition | No Placebo | Postnatal | 274 | 55 | 71 |
| Effects of early prebiotic and probiotic supplementation on development of gut microbiota and fussing and crying in preterm infants: a randomized, double-blind, placebo-controlled trial | Partty, A | 2013 | J Pediatr | Finland | High-Income Country | Both Public and Private | Individual | Drug/  Nutrition | Placebo | Postnatal | 535 | 94 | 23 |
| The NOFLO trial: low-flow nasal prongs therapy in weaning nasal continuous positive airway pressure in preterm infants | O'Donnell, S | 2013 | J Pediatr | Czech Republic,Ireland | High-Income Country | Not Stated | Individual | Medical Device | No Placebo | Postnatal | 96 | 78 | 92 |
| Long-Term Effects of Inhaled Budesonide for Bronchopulmonary Dysplasia | Bassler, D | 2018 | New England Journal of Medicine | Czech Republic,France,Finland,Germany,Israel,Italy,Netherlands,UK | High-Income Country | Both Public and Private | Individual | Drug/  Nutrition | Placebo | Postnatal | 2233 | 863 | 64 |
| Intervention minimizing preterm infants' exposure to NICU light and noise | Aita, M | 2013 | Clinical Nursing Research | Canada | High-Income Country | Public | Individual | Medical Device | No Placebo | Postnatal | 136 | 72 | 64 |
| Comparison of cast materials for the treatment of congenital idiopathic clubfoot using the Ponseti method: a prospective randomized controlled trial | Hui, C | 2014 | Canadian Journal of Surgery | Canada | High-Income Country | Not Stated | Individual | Medical Device | No Placebo | Postnatal | 45 | 30 | 86 |
| Effect of cobedding twins on coregulation, infant state, and twin safety | Hayward, K | 2015 | JOGNN | Canada | High-Income Country | Public | Individual | Other | No Placebo | Postnatal | 320 | 117 | 77 |
| Poractant alfa versus bovine lipid extract surfactant for infants 24+0 to 31+6 weeks gestational age: A randomized controlled trial | Lemyre, B | 2017 | PLoS ONE | Canada | High-Income Country | Both Public and Private | Individual | Drug/  Nutrition | No Placebo | Both Antenatal and Prenatal | 560 | 88 | 47 |
| Effect of Nasal Continuous Positive Airway Pressure (NCPAP) Cycling and Continuous NCPAP on Successful Weaning: A Randomized Controlled Trial | Nair, V | 2015 | Indian J Pediatr | Canada | High-Income Country | Public | Individual | Medical Device | No Placebo | Postnatal | 48 | 30 | 88 |
| Chest Compression During Sustained Inflation and Versus 3:1 Chest Compression: Ventilation Ratio During Neonatal Cardiopulmonary Resuscitation - A Randomized Feasibility Trial | Schmolzer, G | 2017 | Circulation | Canada | High-Income Country | Private | Individual | Other | No Placebo | Postnatal | 11 | 11 | 100 |
| Randomized trial of exclusive human milk versus preterm formula diets in extremely premature infants | Cristofalo, E | 2013 | J Pediatr | Austria,USA | High-Income Country | Not Stated | Individual | Drug/  Nutrition | No Placebo | Postnatal | 67 | 53 | 82 |
| General Movements in preterm infants undergoing craniosacral therapy: a randomised controlled pilot-trial | Raith, W | 2016 | BMC Complementary and Alternative Medicine | Austria | High-Income Country | Not Stated | Individual | Medical Device | No Placebo | Postnatal | 58 | 30 | 81 |
| A randomized controlled trial to compare heated humidified high-flow nasal cannulae with nasal continuous positive airway pressure postextubation in premature infants | Collins, C | 2013 | J Pediatr | Australia | High-Income Country | Not Stated | Individual | Medical Device | No Placebo | Postnatal | 311 | 132 | 86 |
| Mask Versus Nasal Tube for Stabilization of Preterm Infants at Birth: A Randomized Controlled Trial | Kamlin, C | 2013 | Pediatrics | Australia | High-Income Country | Public | Individual | Medical Device | No Placebo | Both Antenatal and Prenatal | 630 | 363 | 98 |
| A randomised placebo-controlled trial of early treatment of the patent ductus arteriosus | Kluckow, M | 2014 | Arch Dis Child Fetal Neonatal Ed | Australia | High-Income Country | Private | Individual | Drug/  Nutrition | Placebo | Postnatal | 393 | 162 | 61 |
| The effect of sildenafil on evolving bronchopulmonary dysplasia in extremely preterm infants: a randomised controlled pilot study | Konig, K | 2014 | J Matern Fetal Neonatal Med | Australia | High-Income Country | Not Stated | Individual | Drug/  Nutrition | Placebo | Postnatal | 206 | 20 | 80 |
| Effect of Bifidobacterium breve M-16V supplementation on fecal bifidobacteria in preterm neonates--a randomised double blind placebo controlled trial | Patole, S | 2014 | PLoS ONE | Australia | High-Income Country | Not Stated | Individual | Drug/  Nutrition | Placebo | Postnatal | 324 | 159 | 98 |
| Novel feeding system to promote establishment of breastfeeds after preterm birth: a randomized controlled trial | Simmer, K | 2016 | Journal of Perinatology | Australia | High-Income Country | Private | Individual | Medical Device | No Placebo | Postnatal | 322 | 100 | 47 |
| A Randomized, Double-Blind, Placebo-Controlled Trial of Pleconaril for the Treatment of Neonates With Enterovirus Sepsis | Abzug, M | 2015 | Journal of the Pediatric Infectious Diseases Society | USA | High-Income Country | Public | Individual | Drug/  Nutrition | Placebo | Postnatal | Not Stated | 31 | Un-available |
| Daily Enteral DHA Supplementation Alleviates Deficiency in Premature Infants | Baack, M | 2016 | Lipids | USA | High-Income Country | Both Public and Private | Individual | Drug/  Nutrition | Placebo | Postnatal | Not Stated | 90 | Un-available |
| Preference for infant-directed speech in preterm infants | Butlera, S | 2014 | Infant Behavior and Development | USA | High-Income Country | Not Stated | Individual | Other | No Placebo | Postnatal | Not Stated | 24 | Un-available |
| Effect of High-Dose Cysteine Supplementation on Erythrocyte Glutathione: A Double-Blinded, Randomized Placebo-Controlled Pilot Study in Critically Ill Neonates | Calkins, K | 2016 | J Parenter Enteral Nutr | USA | High-Income Country | Public | Individual | Drug/  Nutrition | Placebo | Postnatal | Not Stated | 46 | Un-available |
| Randomized controlled trial of vinyl bags versus thermal mattress to prevent hypothermia in extremely low-gestational-age infants | Mathew, B | 2013 | American Journal of Perinatology | USA | High-Income Country | Not Stated | Individual | Medical Device | No Placebo | Antenatal | ?? | 41 | Un-available |
| Preoperative steroid treatment does not improve markers of inflammation after cardiac surgery in neonates: results from a randomized trial | Graham, E | 2014 | J Thorac Cardiovasc Surg | USA | High-Income Country | Not Stated | Individual | Drug/  Nutrition | Placebo | Postnatal | 97 | 78 | Un-available |
| Randomized control trial comparing physiologic effects in preterm infants during treatment with nasal continuous positive airway pressure (NCPAP) generated by Bubble NCPAP and Ventilator NCPAP: a pilot study | Guerin, C | 2016 | J Perinat Med | USA | High-Income Country | Private | Individual | Medical Device | No Placebo | Postnatal | Not Stated | 18 | Un-available |
| Delayed Cord Clamping in Newborns Born at Term at Risk for Resuscitation: A Feasibility Randomized Clinical Trial | Katheria, A | 2017 | Journal of Pediatrics | USA | High-Income Country | Not Stated | Individual | Other | No Placebo | Antenatal | Not Stated | 60 | Un-available |
| Umbilical Cord Milking Versus Delayed Cord Clamping in Preterm Infants | Katheria, A | 2015 | Pediatrics | USA | High-Income Country | Public | Individual | Other | No Placebo | Both Antenatal and Prenatal | 342 | 197 | Un-available |
| Skin-to-skin contact diminishes pain from hepatitis B vaccine injection in healthy full-term neonates | Kostandy, R | 2013 | Neonatal Network | USA | High-Income Country | Private | Individual | Drug/  Nutrition | No Placebo | Postnatal | Not Stated | 36 | Un-available |
| Bi-level CPAP does not improve gas exchange when compared with conventional CPAP for the treatment of neonates recovering from respiratory distress syndrome | Lampland, A | 2015 | Archives of Disease in Childhood | USA | High-Income Country | Private | Individual | Medical Device | No Placebo | Postnatal | Not Stated | 20 | Un-available |
| Soy protein-based infant formulas with supplemental fructooligosaccharides: gastrointestinal tolerance and hydration status in newborn infants | Lasekan, J | 2015 | Nutrients | USA | High-Income Country | Private | Individual | Drug/  Nutrition | No Placebo | Postnatal | Not Stated | 195 | Un-available |
| The effects of music therapy on vital signs, feeding, and sleep in premature infants | Loewy, J | 2013 | Pediatrics | USA | High-Income Country | Private | Individual | Other | No Placebo | Postnatal | Not Stated | 272 | Un-available |
| Effects of cow milk versus extensive protein hydrolysate formulas on infant cognitive development | Mennella, J | 2016 | Amino Acids | USA | High-Income Country | Public | Individual | Drug/  Nutrition | No Placebo | Postnatal | Not Stated | 79 | Un-available |
| Efficacy of clonidine versus phenobarbital in reducing neonatal morphine sulfate therapy days for neonatal abstinence syndrome. A prospective randomized clinical trial | Surran, B | 2013 | Journal of Perinatology | USA | High-Income Country | Private | Individual | Drug/  Nutrition | No Placebo | Postnatal | 146 | 68 | Un-available |
| Besifloxacin Ophthalmic Suspension 0.6% Compared with Gatifloxacin Ophthalmic Solution 0.3% for the Treatment of Bacterial Conjunctivitis in Neonates | Sanfilippo, C | 2017 | Drug/Nutritions R D | USA | High-Income Country | Private | Individual | Drug/  Nutrition | No Placebo | Postnatal | Not Stated | 33 | Un-available |
| Inhaled PGE1 in neonates with hypoxemic respiratory failure: Two pilot feasibility randomized clinical trials | Sood, B | 2014 | Trials | USA | High-Income Country | Not Stated | Individual | Drug/  Nutrition | No Placebo | Postnatal | 46 | 7 | Un-available |
| Conformational positioning improves sleep in premature infants with feeding difficulties | Visscher, M | 2015 | J Pediatr | USA | High-Income Country | Private | Individual | Other | No Placebo | Postnatal | Not Stated | ?? | Un-available |
| Mother-infant interaction improves with a developmental intervention for mother-preterm infant dyads | White-  Traut, R | 2013 | Infant Behavior & Development | USA | High-Income Country | Both Public and Private | Individual | Other | No Placebo | Postnatal | 230 | 198 | Un-available |
| Heated, humidified high-flow nasal cannula versus nasal CPAP for respiratory support in neonates | Yoder, B | 2013 | Pediatrics | USA | High-Income Country | None | Individual | Medical Device | No Placebo | Postnatal | Not Stated | 432 | Un-available |
| Bifidobacterium breve BBG-001 in very preterm infants: a randomised controlled phase 3 trial | Costeloe, K | 2016 | Lancet | UK | High-Income Country | Public | Individual | Drug/  Nutrition | Placebo | Postnatal | Not Stated | 1315 | Un-available |
| Neonatal ECMO study of temperature (NEST): a randomized controlled trial | Field, D | 2013 | Pediatrics | UK | High-Income Country | Private | Individual | Medical Device | No Placebo | Postnatal | Not Stated | 111 | Un-available |
| Proportional assist versus assist control ventilation in premature infants | Shetty, S | 2016 | Eur J Pediatrics | UK | High-Income Country | Both Public and Private | Individual | Medical Device | No Placebo | Postnatal | Not Stated | 8 | Un-available |
| Crossover study of assist control ventilation and neurally adjusted ventilatory assist | Shetty, S | 2017 | Eur J Pediatrics | UK | High-Income Country | Both Public and Private | Individual | Medical Device | No Placebo | Postnatal | Not Stated | ?? | Un-available |
| Comparison of physiological and behavioral responses to fresh and thawed breastmilk in premature infants--a preliminary study | Hung, H | 2013 | Breastfeed Med | Taiwan | High-Income Country | Private | Individual | Drug/  Nutrition | No Placebo | Postnatal | Not Stated | 18 | Un-available |
| Plasma concentrations of levobupivacaine associated with two different intermittent wound infusion regimens following surgical ductus ligation in preterm infants | Anell-Olofsson, M | 2015 | Pediatric Anesthesia | Sweden | High-Income Country | Public | Individual | Drug/  Nutrition | No Placebo | Postnatal | Not Stated | 18 | Un-available |
| A randomised trial of continuous skin-to-skin contact after preterm birth and the effects on salivary cortisol, parental stress, depression, and breastfeeding | Morelius, E | 2015 | Early Human Development | Sweden | High-Income Country | Not Stated | Individual | Other | No Placebo | Postnatal | Not Stated | 42 | Un-available |
| Randomised crossover trial showed that using breast milk or sucrose provided the same analgesic effect in preterm infants of at least 28 weeks | Collados-Gomez, L | 2018 |  | Spain | High-Income Country | Not Stated | Individual | Drug/  Nutrition | No Placebo | Postnatal | 137 | 66 | Un-available |
| Effect of Milking Maneuver in Preterm Infants: A Randomized Controlled Trial | Lago Leal, V | 2018 | Fetal Diagnosis and Therapy | Spain | High-Income Country | Not Stated | Individual | Other | No Placebo | Antenatal | 197 | 138 | Un-available |
| The effects of inhaled albuterol in transient tachypnea of the newborn | Kim, M | 2014 | Allergy Ashtma Immunol Res | South Korea | High-Income Country | Private | Individual | Drug/  Nutrition | Placebo | Postnatal | Not Stated | 28 | Un-available |
| Effect of Saccharomyces boulardii and Mode of Delivery on the Early Development of the Gut Microbial Community in Preterm Infants | Zeber-Lubecka, N | 2016 | PLoS ONE | Poland | High-Income Country | Public | Individual | Drug/  Nutrition | Placebo | Postnatal | Not Stated | 55 | Un-available |
| Maternal Holding vs Oral Glucose Administration as Nonpharmacologic Analgesia in Newborns: A Functional Neuroimaging Study | Bembich, S | 2015 | JAMA Pediatrics | Italy | High-Income Country | Private | Individual | Drug/  Nutrition | No Placebo | Postnatal | Not Stated | 40 | Un-available |
| Semipermeable membranes and hypernatremic dehydration in preterms. A randomized-controlled trial | Cardiello, V | 2018 | Early Human Development | Italy | High-Income Country | None | Individual | Medical Device | No Placebo | Postnatal | Not Stated | 164 | Un-available |
| A multicenter, randomised, controlled trial of osteopathic manipulative treatment on preterm infants | Cerritelli, F | 2014 | Archives of Disease in Childhood | Italy | High-Income Country | None | Individual | Other | No Placebo | Postnatal | 1169 | 720 | Un-available |
| No effect of adding dairy lipids or long chain polyunsaturated fatty acids on formula tolerance and growth in full term infants: a randomized controlled trial | Gianni, M | 2018 | BMC Pediatrics | Italy | High-Income Country | Private | Individual | Drug/  Nutrition | No Placebo | Postnatal | Not Stated | 117 | Un-available |
| Growth and safety evaluation of infant formulae containing oligosaccharides derived from bovine milk: a randomized, double-blind, noninferiority trial | Meli, F | 2014 | BMC Pediatrics | Italy | High-Income Country | Private | Individual | Drug/  Nutrition | No Placebo | Postnatal | Not Stated | 311 | Un-available |
| Clinical evaluation of two different protein content formulas fed to full-term healthy infants: a randomized controlled trial | Nadia, L | 2018 | BMC Pediatrics | Italy | High-Income Country | Private | Individual | Drug/  Nutrition | No Placebo | Postnatal | 274 | 168 | Un-available |
| Music reduces pain perception in healthy newborns: A comparison between different music tracks and heartbeat | Rossi, A | 2017 | Developmental Medicine and Child Neurology | Italy | High-Income Country | Not Stated | Individual | Other | No Placebo | Postnatal | Not Stated | 80 | Un-available |
| A randomised crossover study of low-flow air or oxygen via nasal cannulae to prevent desaturation in preterm infants | Hensey, C | 2013 | Arch Dis Child Fetal Neontal | Ireland | High-Income Country | Not Stated | Individual | Medical Device | No Placebo | Postnatal | Not Stated | 14 | Un-available |
| Gut microbiota analysis reveals a marked shift to bifidobacteria by a starter infant formula containing a synbiotic of bovine milk-derived oligosaccharides and Bifidobacterium animalis subsp. lactis CNCM I-3446 | Simeoni, U | 2016 | Environ-mental Micro-biology | France,  Germany,Poland,  Switzer-land | High-Income Country | Private | Individual | Drug/  Nutrition | No Placebo | Postnatal | Not Stated | 115 | Un-available |
| Neurally adjusted ventilatory assist (NAVA) in preterm newborn infants with respiratory distress syndrome-a randomized controlled trial | Kallio, M | 2016 | Eur J Pediatr | Finland | High-Income Country | Private | Individual | Medical Device | No Placebo | Postnatal | Not Stated | 60 | Un-available |
| Adding hydrocortisone as 1st line of inotropic treatment for hypotension in very low birth weight infants | Hochwald, O | 2014 | Indian Jounal of Pediatrics | Canada | High-Income Country | None | Individual | Drug/  Nutrition | Placebo | Postnatal | Not Stated | 22 | Un-available |
