# Supplementary material for: Informed consent rates for neonatal randomized controlled trials in low- and lower middle-income versus high-income countries: A systematic review
Source: PLoS One. 2021 Mar 9;16(3):e0248263. doi: 10.1371/journal.pone.0248263 (PMC7943024; doi:10.1371/journal.pone.0248263)
Supplement: S5 Table — (DOCX) [file pone.0248263.s006.docx]

**S5 Table. Consent rates by country**

|  | **Total trials, N** | **Trials with**  **consent rate, n** | **Median % consented (IQR)** |
| --- | --- | --- | --- |
| ***LMICs*** | | | |
| Armenia | 1 | 1 | 96.9 (.) |
| Bangladesh | 3 | 2 | 85.3 (73.6-97.1) |
| Bolivia | 1 | 1 | 99.2 (.) |
| Cameroon | 1 | 0 | . |
| Egypt | 18 | 9 | 87.8 (85.3-95.3) |
| Gambia | 1 | 0 | . |
| Ghana | 1 | 0 | . |
| Guinea-Bissau | 2 | 2 | 99.9 (99.8-100.0) |
| India | 135 | 106 | 95.8 (88.9-98.7) |
| Indonesia | 4 | 2 | 97.2 (95.2-99.3) |
| Jordan | 2 | 2 | 93.2 (91.7-94.8) |
| Nepal | 2 | 1 | 66.4 (.) |
| Nigeria | 1 | 0 | . |
| Pakistan | 12 | 5 | 89.6 (88.4-95.3) |
| Papua New Guinea | 1 | 0 | . |
| Philippines | 1 | 0 | . |
| Tanzania | 1 | 1 | 98.6 (.) |
| Uganda | 3 | 2 | 100.0 (100.0-100.0) |
| Vietnam | 1 | 0 | . |
| Zambia | 2 | 1 | 97.0 (.) |
| ***HICs*** | | | |
| Australia | 6 | 6 | 83.1 (61.4-97.8) |
| Austria | 1 | 1 | 81.1 (.) |
| Canada | 7 | 6 | 81.6 (64.3-88.2) |
| Finland | 2 | 1 | 23.2 (.) |
| France | 1 | 1 | 70.5 (.) |
| Germany | 2 | 2 | 88.6 (81.6-95.7) |
| Greece | 1 | 1 | 72.2 (.) |
| Hong Kong | 1 | 1 | 90.9 (.) |
| Ireland | 3 | 2 | 88.5 (86.3-90.6) |
| Israel | 1 | 1 | 97.4 (.) |
| Italy | 16 | 9 | 94.2 (93.0-97.4) |
| Netherlands | 4 | 4 | 59.2 (41.6-77.6) |
| New Zealand | 2 | 2 | 80.3 (75.4-85.2) |
| Norway | 1 | 1 | 98.0 (.) |
| Poland | 1 | 0 | . |
| South Korea | 2 | 1 | 81.3 (.) |
| Spain | 4 | 2 | 86.1 (82.7-89.5) |
| Sweden | 2 | 0 | . |
| Taiwan | 1 | 0 | . |
| United Kingdom | 8 | 4 | 61.3 (39.8-76.5) |
| United States | 37 | 17 | 69.5 (65.7-86.4) |
| Multi-country: Austria, United States | 1 | 1 | 81.5 (.) |
| Multi-country: France, Germany, Poland, Switzerland | 1 | 0 | . |
| Multi-country: Czech Republic, Ireland | 1 | 1 | 91.8 (.) |
| Multi-country: Czech Republic, France, Finland, Germany, Israel, Italy, Netherlands, United Kingdom | 1 | 1 | 64.3 (.) |
