# Supplementary material for: Informed consent rates for neonatal randomized controlled trials in low- and lower middle-income versus high-income countries: A systematic review
Source: PLoS One. 2021 Mar 9;16(3):e0248263. doi: 10.1371/journal.pone.0248263 (PMC7943024; doi:10.1371/journal.pone.0248263)
Supplement: S6 Table — (DOCX) [file pone.0248263.s007.docx]

**S6 Table. Odds of obtaining a consent rate (in article or by email response from study author) in LMIC versus HIC trials**

| **Control-intervention trial type** | **Unadjusted OR**  **(95% CI)** | **p-value** | **Adjusted OR**  **(95% CI)** | **p-value** |
| --- | --- | --- | --- | --- |
| Placebo–drug/nutrition | 1.79 (0.54–5.89) | 0.3407 | 1.64 (0.46–5.87) | 0.4445 |
| No placebo–drug/nutrition | 1.48 (0.65–3.39) | 0.3522 | 0.89 (0.35–2.28) | 0.8128 |
| No placebo–medical device | 1.75 (0.60–5.12) | 0.3048 | 1.33 (0.42–4.25) | 0.6257 |
| No placebo–other | 1.33 (0.48–3.72) | 0.5825 | 1.01 (0.33–3.08) | 0.9905 |
| Note: N=300 observations for both models. Unadjusted model with an LR Chi-Square of 4.55 (p=0.7152) and Pseudo R-square of 0.0119. Adjusted model derived from logistic regression adjusting for funding, timing of consent, publication year and log number enrolled with an LR Chi-Square of 16.46 (p=0.3523) and Pseudo R-square of 0.0431. | | | | |
