# Supplementary material for: Informed consent rates for neonatal randomized controlled trials in low- and lower middle-income versus high-income countries: A systematic review
Source: PLoS One. 2021 Mar 9;16(3):e0248263. doi: 10.1371/journal.pone.0248263 (PMC7943024; doi:10.1371/journal.pone.0248263)
Supplement: Attachment — Submitted filename: Response Letter PLOS One 2-5-21.docx [file pone.0248263.s010.docx]

**DIVISION OF NEONATAL-PERINATAL MEDICINE**

**THE UNIVERSITY OF NORTH CAROLINA AT CHAPEL HILL**

**CB# 7596, 4^th^ Floor, UNC Hospitals**

**Chapel Hill, NC 27599-7596**

February 5, 2021

Ghislaine JMW van Thiel
Academic Editor
PLOS ONE

Dear Dr. van Thiel,

Thank you for your consideration of our manuscript titled *Informed consent rates for neonatal randomized controlled trials in low- and lower middle-income versus high-income countries: A systematic review*. We thank the editor and the reviewers for their helpful comments, and respectfully submit our revised manuscript. Below is a point-by-point response to the comments received, with our responses in *blue italics*.

Sincerely,

Jacquelyn Patterson, MD, MPH

Assistant Professor of Pediatrics

**Journal requirements**

1. Please ensure that your manuscript meets PLOS ONE's style requirements, including those for file naming. The PLOS ONE style templates can be found at

<https://journals.plos.org/plosone/s/file?id=wjVg/PLOSOne_formatting_sample_main_body.pdf> and

<https://journals.plos.org/plosone/s/file?id=ba62/PLOSOne_formatting_sample_title_authors_affiliations.pdf>

*We have formatted our manuscript according to the PLOS One style requirements, including those for file naming.*

2. Please consider discussing the quality of each of the studies included in this review and their biases. In addition, please update your search to allow the inclusion of studies published in the past 12 months.

*We did not assess the quality of the studies included since the quality of a study does not impact the validity of the informed consent rate reported. As such, traditional bias assessments were not relevant for this review of informed consent rates.*

*We agree that it is important for systematic reviews and meta-analyses to be contemporary, ideally published within one year of the search date. This is to ensure there are no newly published trials which would influence the results of the meta-analysis, such as when reviewing the efficacy of a drug or prognostic accuracy of a test.*

*In the case of this systematic review, we focused on all trials published within a particular time period, requiring us to screen approximately 3,500 trials and to extract data from approximately 200 full text articles. As such, this systematic review took us approximately 2 years to complete. Given the additional 6 months our publication has been under review at PLOS One, our search is now approximately 2.5 years old. Updating our search to include the past 2.5 years of trials will take at least one year to perform.*

*To address the editor’s concern and to demonstrate that our review is sufficiently contemporary, we repeated the search and extracted consent rates in a random sample of 20 trials published in 2020. Our results demonstrate that consent rates have not changed since the end date of our previous search, and that the conclusions of our systematic review remain valid. We have included this data in both the methods and results section of the revised manuscript.*

*In lines 91-93 and 130-132 of the methods, the revised manuscript includes the following new text:*

*“In January 2021, we repeated the literature search using the same search strategy and retrieved a random sample of 20 neonatal RCTs (10 trials from LMICs and 10 trials from HICs) published between January 1, 2020 and January 1, 2021. To explore whether our findings remain relevant in newly published trials, we extracted and analyzed consent rates only from the random sample of trials obtained from our more recent search.”*

*Additionally, in table 2 under ‘Note’ in the results (lines 214-216), the revised manuscript includes the following new text:*

*“In our random sample of neonatal RCTs published between January 2020 and January 2021, the median percent consented in LMICs was 95.9 (IQR 94.4-96.1) whereas the median percent consented in HICs was 77.0 (IQR 66.5-87.5).”*

3. We note that you have stated that you will provide repository information for your data at acceptance. Should your manuscript be accepted for publication, we will hold it until you provide the relevant accession numbers or DOIs necessary to access your data. If you wish to make changes to your Data Availability statement, please describe these changes in your cover letter and we will update your Data Availability statement to reflect the information you provide.

*We would like to change our Data Availability statement so that it indicates that all data is provided as part of this publication. In this revision, we have included a csv file of our raw data as Supporting Information.*

4. We note that Figure 2 in your submission contain map images which may be copyrighted. All PLOS content is published under the Creative Commons Attribution License (CC BY 4.0), which means that the manuscript, images, and Supporting Information files will be freely available online, and any third party is permitted to access, download, copy, distribute, and use these materials in any way, even commercially, with proper attribution. For these reasons, we cannot publish previously copyrighted maps or satellite images created using proprietary data, such as Google software (Google Maps, Street View, and Earth). For more information, see our copyright guidelines: <http://journals.plos.org/plosone/s/licenses-and-copyright>.

We require you to either (1) present written permission from the copyright holder to publish these figures specifically under the CC BY 4.0 license, or (2) remove the figures from your submission:

*The map in Figure 2 of our manuscript was produced using R version 4.0 and the packages rnaturalearth and ggplot2. As such, copyrighting does not apply.*

5. Please include captions for your Supporting Information files at the end of your manuscript, and update any in-text citations to match accordingly. Please see our Supporting Information guidelines for more information: [http://journals.plos.org/plosone/s/supporting-information](about:blank).

*We have updated our manuscript accordingly.*

6. If applicable, we recommend that you deposit your laboratory protocols in protocols.io to enhance the reproducibility of your results. Protocols.io assigns your protocol its own identifier (DOI) so that it can be cited independently in the future. For instructions see: <http://journals.plos.org/plosone/s/submission-guidelines#loc-laboratory-protocols>

*At the time of acceptance to PLOS One, we will upload our protocol to this repository.*

7. While revising your submission, please upload your figure files to the Preflight Analysis and Conversion Engine (PACE) digital diagnostic tool, <https://pacev2.apexcovantage.com/>. PACE helps ensure that figures meet PLOS requirements. To use PACE, you must first register as a user. Registration is free. Then, login and navigate to the UPLOAD tab, where you will find detailed instructions on how to use the tool. If you encounter any issues or have any questions when using PACE, please email PLOS at [figures@plos.org](mailto:figures@plos.org). Please note that Supporting Information files do not need this step.

*We have uploaded all figure files to PACE.*

**Comments to the Author**

Thank you for the opportunity to review this interesting manuscript reporting the differences in consent rates between LMIC and HIC for trials conducted with neonatal populations. This review is novel and important to inform research decisions. The manuscript is well written with comprehensive tables and figures supporting their analyses and conclusions. There are a few questions and concerns.

1. Please justify why middle-to-high income countries were excluded.

*Our hypothesis that prompted this systematic review was that consent rates in randomized controlled trials conducted in LMICs are much higher than in HICs. Unlike in HICs, LMICs often lack adequate research governance systems to protect trial participants. The situation in upper middle-income countries (UMICs) is much more variable, and the proportion of RCTs conducted in these settings was relatively small. As such, we elected to exclude UMICs from this review.*

2. Was there a difference in rates of consent if mother vs father of the infant consented?

*Details regarding the consent process, including whether the mother or father provided consent, were limited in the articles included. As such, the available data does not allow us to answer this question.*

3. Similar to HIC, please provide acronym for LMIC early in the manuscript and use consistently.

*We provide an acronym for LMIC at its first use in the introduction, and have double checked that it is used consistently throughout the manuscript.*

4. Table 1. I struggled to find which study characteristics were significantly different. It would help the reader if a column with p values was added.

*We have added a column of p-values.*

5. Except for a statement on line 299, the authors are largely silent on monetary and

other incentives to participate in each study. Incentives may be critical to decisions to participate in trials, particularly in LMIC. If data were available for even a subset of included articles, consent by incentives this should be included as sub-analysis. If not, please address as a limitation of the review.

*We agree that incentives may be one reason for higher consent rates in LMICs; we addressed this in lines 293-295 of the discussion, which read as follows:*

*“Furthermore, even with standard of care or placebo control arms, participants may perceive additional incentives such as free health care, increased surveillance and reimbursement of travel costs as benefits to their participation. These incentives may hold greater importance as positive motivators for consent in LMICs.”*

*Per your suggestion, we have also added this to the limitations of our study on lines 311-313:*

*“We cannot comment on other factors beyond trial characteristics that have been associated with informed consent rates such as parental characteristics, physicians approaching participants for consent, or incentives to participation.”*

6. There may be a missing reference on line 285.

*Thank you for pointing this out. We have double checked that all intended references were included and deleted “[ref]” in the text.*

**North Carolina Children's Hospital ▪ University of North Carolina Hospitals**

**Newborn Critical Care Center**

**Office: (919) 966-5063 · Fax: (919) 966-3034**

**Website: http://pediatrics.med.unc.edu/pediatric-specialties/neonatal-perinatal-medicine**
